# Supplementary figures and images for: Kaposi’s sarcoma-associated herpesvirus (KSHV) utilizes the NDP52/CALCOCO2 selective autophagy receptor to disassemble processing bodies
Source: PLoS Pathog. 2023 Jan 12;19(1):e1011080. doi: 10.1371/journal.ppat.1011080 (PMC9876383; doi:10.1371/journal.ppat.1011080)

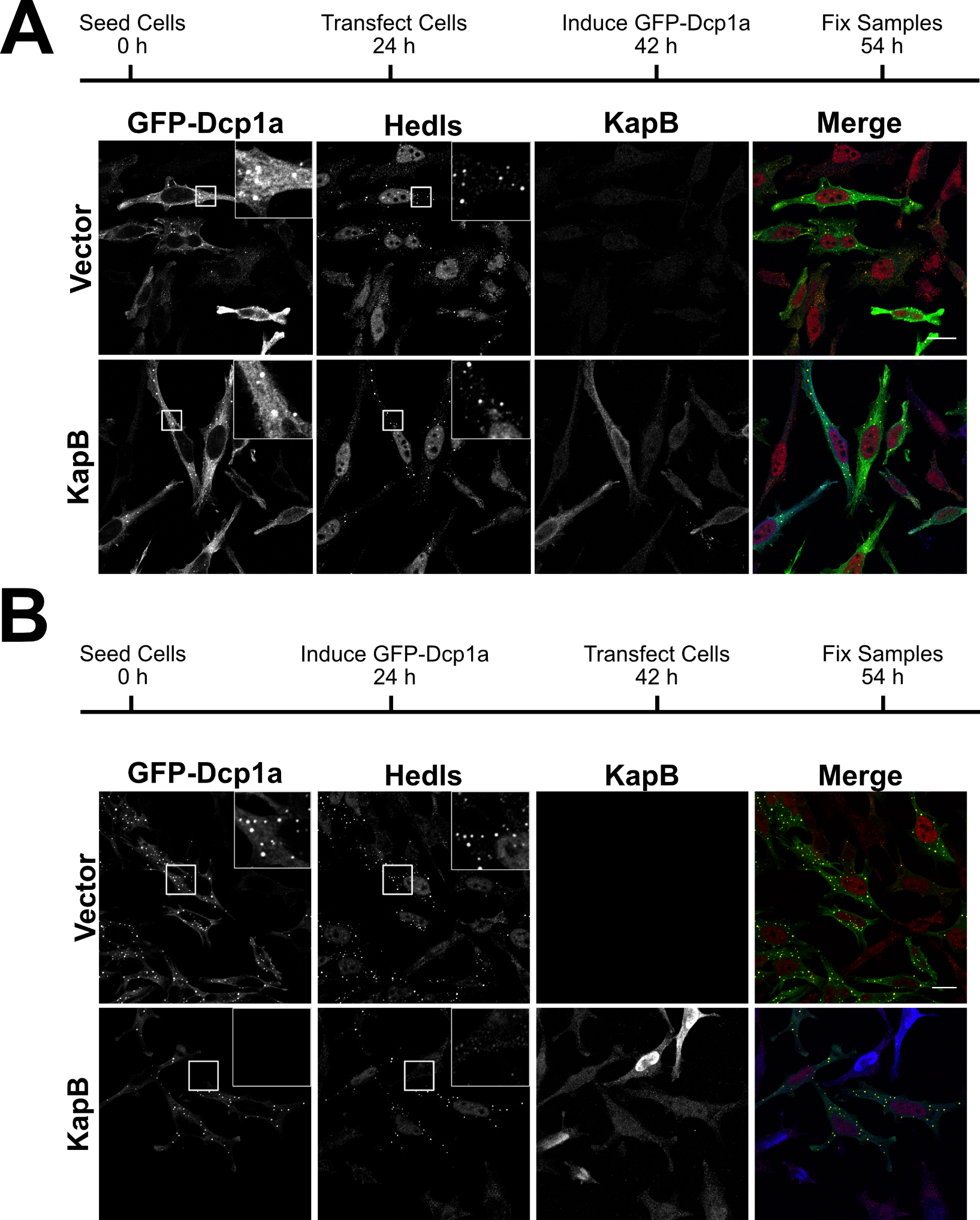

Supplement: S1 Fig — HeLa cells expressing a doxycycline-inducible GFP-Dcp1a were used to determine whether KapB expression prevented granule assembly or promoted disassembly. A: HeLa cells were transfected with either KapB or an empty vector control prior to inducing expression of GFP-Dcp1a with doxycycline (1 μg/mL). Cells were fixed 12 h post-induction and immunostained for Hedls (PBs, red) and KapB (blue). Scale bar = 20 μm. B: GFP-Dcp1a expression was induced with doxycycline (1 μg/mL) in HeLa cells prior to transfection with either KapB or an empty vector control. Cells were fixed 12 h post-transfection and immunostained for Hedls (PBs, red) and KapB (blue). Scale bar = 20 μm. (TIFF) [file ppat.1011080.s001.tiff]

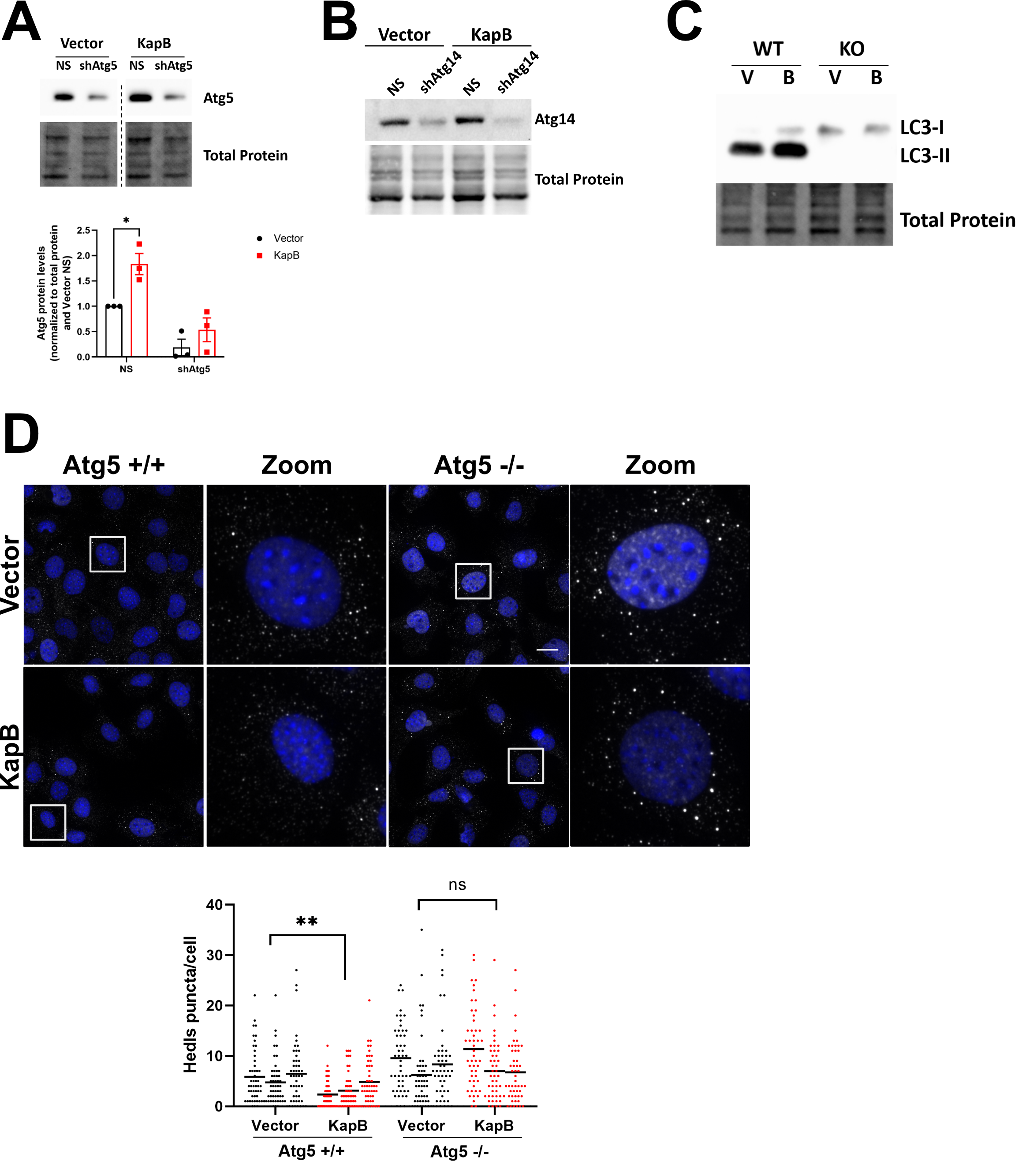

Supplement: S2 Fig — A&B: HUVECs were sequentially transduced: first with recombinant lentiviruses expressing either shRNAs targeting Atg5 or Atg14 (shAtg5, shAtg14) or a non-targeting control (NS) and selected with puromycin (1 μg/mL), and second with either KapB or an empty vector control and selected with blasticidin (5 μg/mL). Samples were lysed in 2X Laemmli and resolved by SDS-PAGE. Samples were immunoblotted for Atg5 (A) or Atg14 (B). For Atg5 (A), representative blot is from the same membrane, hashed line indicates skipped lanes. For Atg5 (A) samples were quantified by normalizing Atg5 protein levels to the total protein in each lane using Image Lab (BioRad) and then to Vector NS. Results were plotted in GraphPad, a 2-way ANOVA was performed, ±SEM; n = 3, *** = P<0.001. C: Atg5 +/+ (WT) and -/- (KO) MEFs were transduced as in A and harvested in 2X Laemmli buffer before being resolved by SDS-PAGE. Samples were probed for LC3, a representative blot is shown. D: Atg5 +/+ and -/- MEFs were transduced with recombinant viruses expressing KapB or an empty vector control and selected with blasticidin (5 μg/mL). Samples were fixed in 4% paraformaldehyde and permeabilized in 0.1% Triton X-100. Immunofluorescence was performed for Hedls (PBs, white) and DAPI (nuclei, blue). Scale bar = 20 μm. Hedls puncta were quantified using CellProfiler and presented as number of Hedls puncta per cell, all cells counted are displayed. Results were plotted in GraphPad and a 2-way ANOVA was performed on the main column effects with a Tukey’s multiple comparison test, bar represents the mean; n = 3, ** = P<0.01. (TIFF) [file ppat.1011080.s002.tiff]

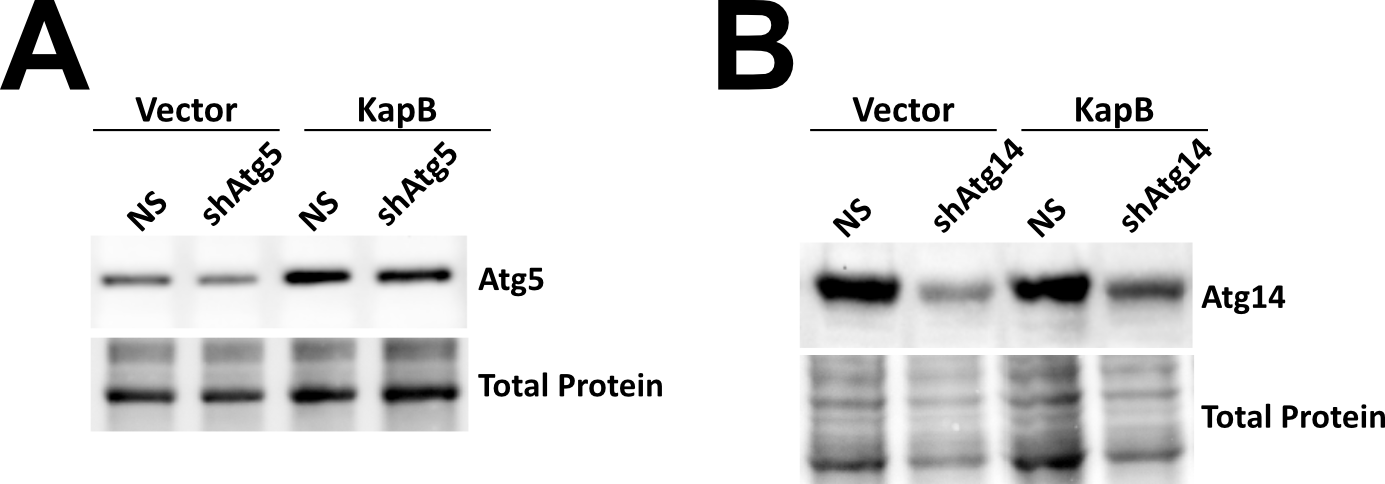

Supplement: S3 Fig — HeLa cells were transduced with recombinant lentiviruses expressing either shRNAs targeting Atg5 or Atg14 (shAtg5, shAtg14) or a non-targeting control (NS) and selected with puromycin (1 μg/mL) prior to transfection with KapB or an empty vector for luciferase assays. Samples were lysed in 2X Laemmli buffer and resolved by SDS-PAGE before immunoblotting with Atg5 (A) or Atg14 (B). Representative blots are shown. (TIFF) [file ppat.1011080.s003.tiff]

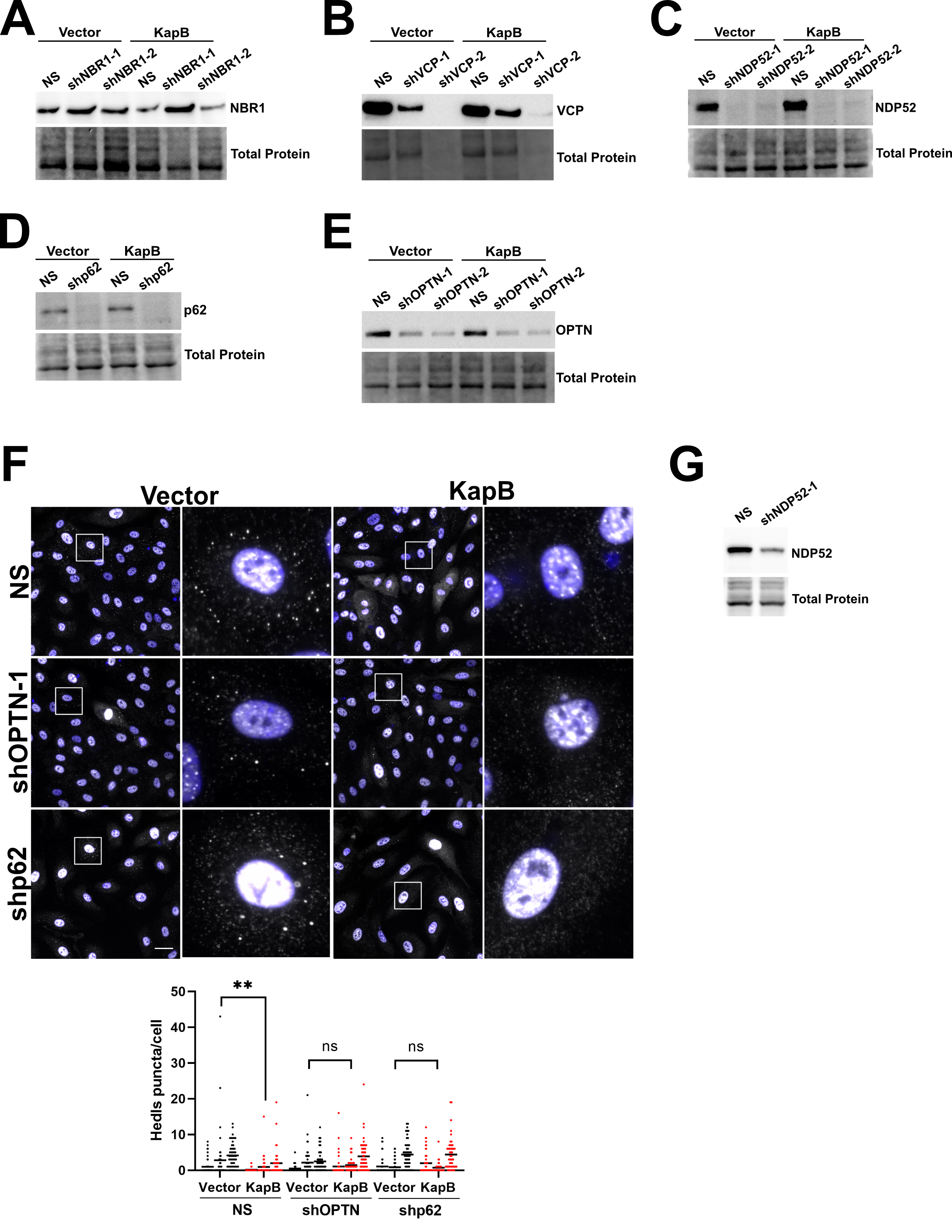

Supplement: S4 Fig — A&B: HUVECs were transduced with shRNAs targeting VCP, NBR1, or a non-targeting control (NS) and selected with puromycin (1 μg/mL), then transduced with an empty vector control or KapB and selected with blasticidin (5 μg/mL). Samples were lysed in 2X Laemmli buffer, resolved by SDS-PAGE, and immunoblotted for NBR1 (A) and VCP (B). Representative blots are shown. Successful VCP knockdown was lethal while NBR1 knockdown was not successful. C: HUVECs were transduced with shRNAs targeting NDP52 or a non-targeting control (NS) and selected with puromycin (1 μg/mL), then transduced with an empty vector control or KapB and selected with blasticidin (5 μg/mL). Samples were lysed in 2X Laemmli buffer, resolved by SDS-PAGE, and immunoblotted for NDP52. D&E: HUVECs were transduced with shRNAs targeting p62, OPTN, or a non-targeting control (NS) and selected with puromycin (1 μg/mL), then transduced with an empty vector control or KapB and selected with blasticidin (5 μg/mL). Samples were lysed in 2X Laemmli buffer and resolved by SDS-PAGE, and immunoblotted for p62 (D) or OPTN (E). Representative blots are shown. F: HUVECs were transduced as in D and E. Coverslips were fixed in 4% paraformaldehyde, permeabilized in 0.1% Triton X-100 and immunostained for Hedls (PBs; white), DAPI (nuclei, blue). Scale bar = 20 μm. Hedls puncta were quantified using CellProfiler and presented as number of Hedls puncta per cell, all cells counted are displayed. Results were plotted in GraphPad and a 2-way ANOVA was performed on the main column effects with a Tukey’s multiple comparison test, bar represents the mean; n = 3, ** = P<0.01. G: HUVECs were transduced with recombinant lentiviruses targeting NDP52 or a non-targeting control (NS) and selected with puromycin (1 μg/mL). Samples were lysed in 2X Laemmli buffer, resolved by SDS-PAGE, and immunoblotted for NDP52 to confirm knockdown for Torin immunofluorescence experiments performed in parallel. (TIFF) [file ppat.1011080.s004.tiff]

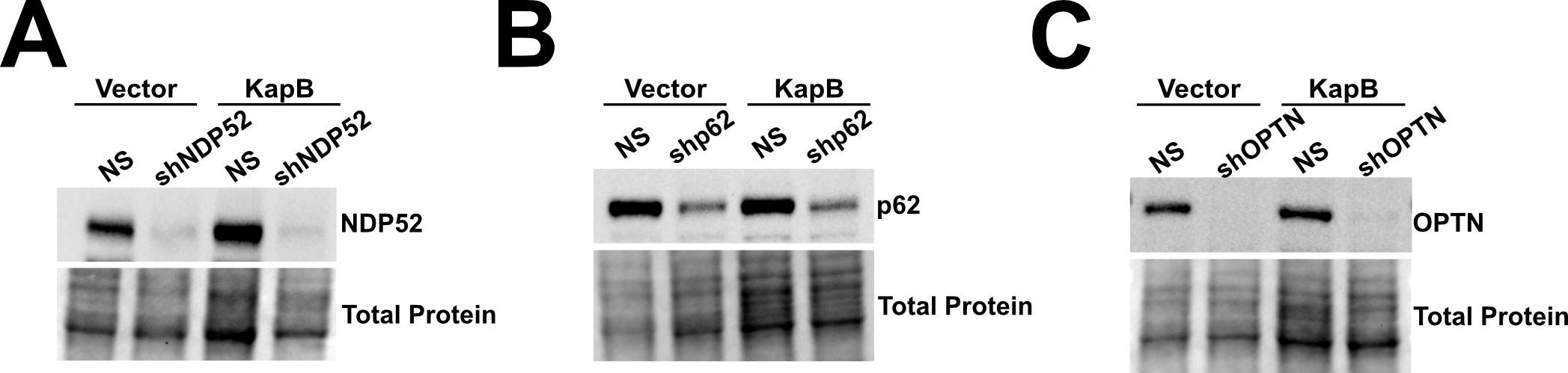

Supplement: S5 Fig — HeLas were transduced with shRNAs targeting NDP52, OPTN, p62, or a non-targeting control (NS) and selected with puromycin (1 μg/mL) prior to transfection with KapB or an empty vector for luciferase assays. Samples were lysed in 2X Laemmli buffer, resolved by SDS-PAGE, and immunoblotted for NDP52 (A), p62 (B), and OPTN (C). Representative blots are shown. (TIFF) [file ppat.1011080.s005.tiff]

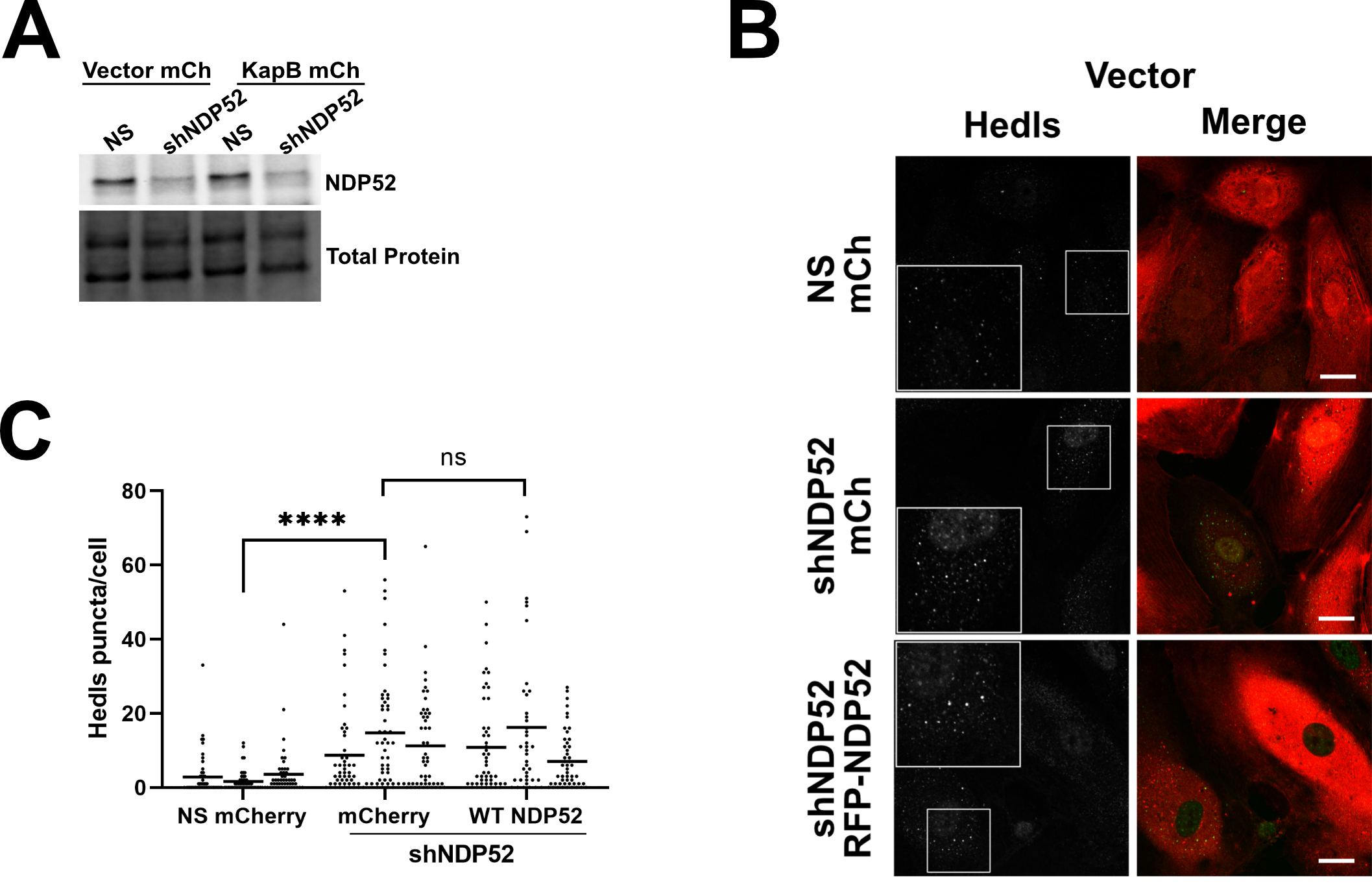

Supplement: S6 Fig — A: Representative western blot of HUVECs transduced with an shRNA targeting the 3’UTR of NDP52 or a non-targeting control (NS) and selected with puromycin (1 μg/mL), then co-transduced with an empty vector control and mCherry (mCh) or KapB and mCh. Samples were lysed in 2X Laemmli, resolved by SDS-PAGE, and immunoblotted for NDP52. B: HUVECs were sequentially transduced first with recombinant lentivirus expressing shNDP52 targeting the 3’-UTR of NDP52 or a NS control and selected with blasticidin (5 μg/mL), and second, with vector and either an mCherry control (mCh) or RFP-NDP52. Coverslips were fixed with 4% paraformaldehyde, permeabilized with 0.1% Triton X-100, and were immunostained with Hedls (PBs, green). Scale bar = 20 μm. C: Hedls puncta were quantified using CellProfiler and presented as number of Hedls puncta per cell, all cells counted are displayed. Results were plotted in GraphPad and a 2-way ANOVA was performed on the main column effects with a Tukey’s multiple comparison test, bar represents the mean; n = 3. (TIFF) [file ppat.1011080.s006.tiff]

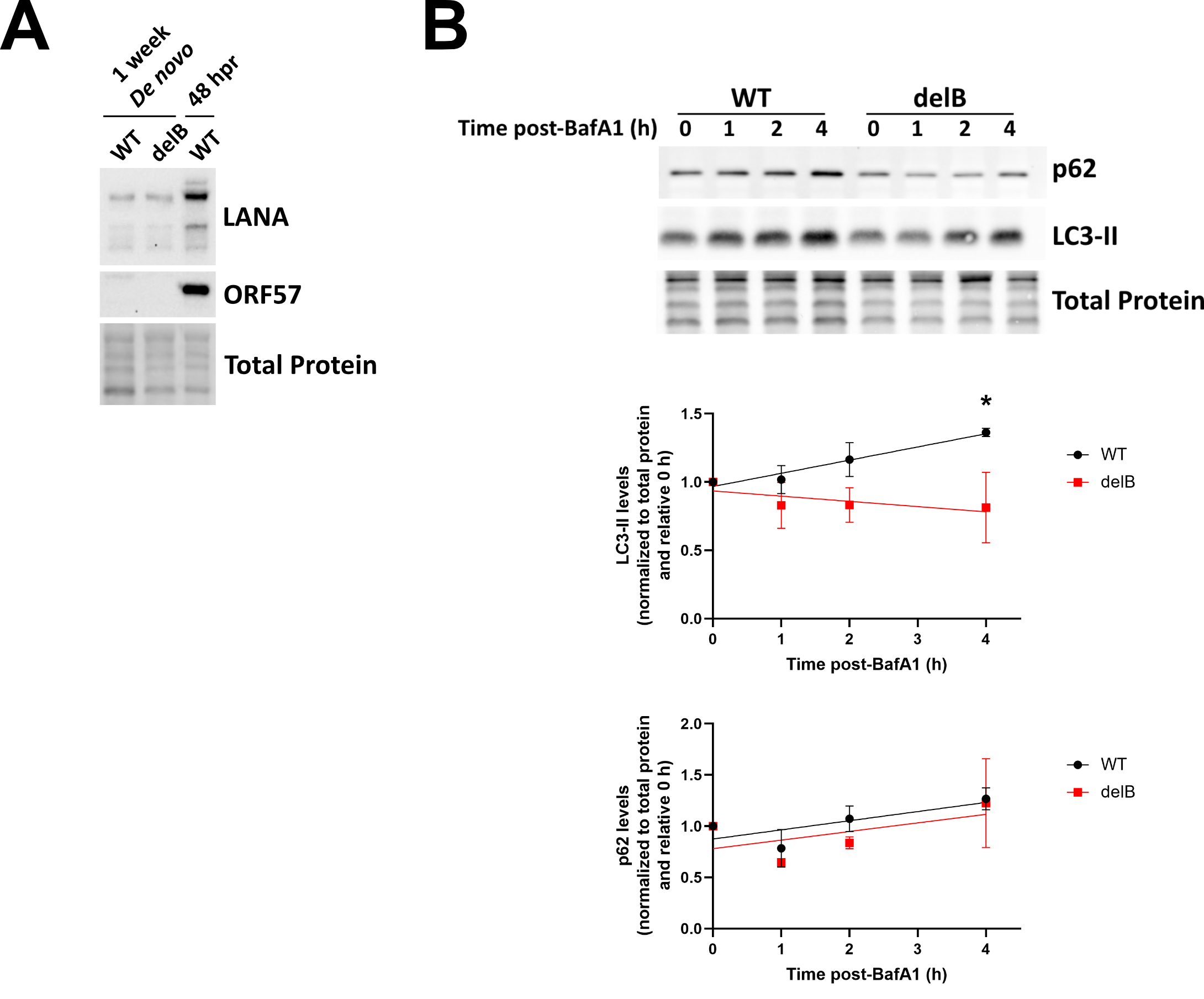

Supplement: S7 Fig — A-B: Naïve iSLK cells were infected with wild-type (WT) or delete KapB (delB) BAC16 KSHV via spinoculation, robust and stable latency was achieved by expanding and selecting cells in hygromycin B (500 μg/mL and then 1200 μg/mL) for one week before seeding for experiments. A: After one week, cells were either lysed for immunoblotting or treated with 1 μg/mL Dox to induce reactivation (WT only) and lysed for immunoblotting 48 h after reactivation. One representative immunoblot of three independent experiments is shown. B: Cells were treated with Bafilomycin A1 (BafA1, 10 nM) or a vehicle control (DMSO) for the indicated times prior to harvest in 2X Laemmli buffer. Protein lysates were resolved by SDS-PAGE and immunoblot was performed for p62 and LC3. Samples were quantified using Image Lab (BioRad) software and then normalized, first to total protein and then to their respective starting time points (0 h). Results were plotted in GraphPad and a linear regression statistical test was performed, ±SEM; n = 3, * = P<0.05. (TIFF) [file ppat.1011080.s007.tiff]
