# Supplementary material for: Kaposi’s sarcoma-associated herpesvirus (KSHV) utilizes the NDP52/CALCOCO2 selective autophagy receptor to disassemble processing bodies
Source: PLoS Pathog. 2023 Jan 12;19(1):e1011080. doi: 10.1371/journal.ppat.1011080 (PMC9876383; doi:10.1371/journal.ppat.1011080)
Supplement: Attachment — Submitted filename: Corcoran Response to Reviewers.pdf [file ppat.1011080.s008.pdf]

Response to Reviewers

Editor:
While some of the reviewers appreciated the potential importance of the findings, there were serious concerns about the physiological relevance of transfection experiments without direct evidence in the context of viral infection.

We appreciate the editor's concern about the biological relevance of our experiments to actual KSHV infection. (A point of clarification – throughout the paper, we used lentiviral transduction of primary endothelial cells to overexpress KapB or to deliver any other genes/shRNAs). To address this overarching concern, we have added several new pieces of data that use virus infection to the manuscript. To summarize these additions, we have added:
-new data (Figure 6A) to show that primary infection of HUVECs with KSHV induces autophagic flux and new data to show that KSHV infection induced the phosphorylation of Ser90 of Beclin-1 (Figure 6B), recapitulating the effect we previously observed with KapB expression
-new data showing that infection with a KSHV recombinant virus that does not produce KapB (delB BAC16) fails to increase autophagic flux and fails to phosphorylate Ser90 of Beclin-1 (Figure 6C-D). (We previously showed that ΔKapB fails to induce PB disassembly – please see Fig 9 of Kleer et al. 2021)
-in consideration of concerns about the variability of our data, we have re-graphed every single piece of data for PB quantification (Figs 1, 2, 5, 7, S2, S4, S6) to show individual data points where each data point represents the number of PBs counted for each cell. These data now provide editors and reviewers with a transparent view of PB count variation between independent experiments and in so doing, also increased the statistical significance of our experiments.

Reviewer #1:

Robinson and colleagues reported that the KSHV protein KapB induces the disappearance of processing bodies (PB) by activating cellular autophagy in the present manuscript. The authors also noted that Kap B-mediated disappearance of PB depends on the cellular genes Atg5 and Atg14. The authors finally reported that during KSHV infection, PB disassembly requires Atg5 and the autophagy receptor NDP2. Based on the data, the authors concluded that PB disassembling through autophagy is an important mechanism to increase the expression of pro-inflammatory genes. KSHV is an important pathogen that is causatively associated with several malignancies. Understanding how KSHV induces an inflammatory environment that supports cancer lesions' onset and progression is highly significant. Therefore, the data reported in this works are of high interest. The strength of this manuscript is the use of several approaches to demonstrate the effect of KapB on PB disassembling, including the use of mutant constructs and different drugs. However, most of the experiments are conducted under overexpression conditions in uninfected cells. Repeating some of the observations in the context of KSHV infection would strengthen the conclusions and increase the significance of the reported data

We thank Reviewer 1 for their appreciation of the significance of these data to KSHV infection. We have taken Reviewer 1's recommendation and repeated several experiments in the context of KSHV infection, as summarized below.

Major Concern:
Most of the experiments exploring the KapB/autophagy axis were performed using ectopic expression of KapB. It would be essential to show that under physiological conditions, i.e., during KSHV infection, KapB/autophagy axis is also operating. For example, in figure 7, during KSHV infection, can depletion of Atg5 or NDP52 affect the expression of ARE-mRNAs and production of inflammatory molecules, as observed in Figure 3?

We appreciate this insightful comment from Reviewer 1. The current manuscript provides data to support that silencing of Atg5 and NDP52 reverses KSHV-mediated PB disassembly (Fig 7A-B of the revised paper). These data show that in control cells, KSHV infection decreases PBs; however, in silenced cells, KSHV infection has no effect on PBs relative to the uninfected matched control. We note that it is not appropriate to directly compare KSHV-infected silenced cells to control cells because silencing autophagy regulatory proteins impacts PB baseline levels. We have added an addition description of these data in Lines 592-600 of the revised manuscript.

Minor Concerns:

In Figure 1D, the authors indicated that depletion of Atg14 restored PBs in HUVEC cells expressing KapB protein but did not affect control cells. It seems instead that also in control cells, the lack of Atg14 restored PBs. There is no statistical analysis for the increase observed in the control cells (black dots) NS compared to shAtg14. The authors should add it.

Ribonucleoprotein granules require mechanisms to control their assembly and their disassembly. We believe we have uncovered, using a viral protein, a cellular mechanism that exists to control PB disassembly – the 'off' switch for PBs. Our model is that NDP52- dependent autophagic turnover of PBs normally occurs at a baseline level (like all autophagic processes) and this turnover is accelerated or enhanced when KapB is expressed. For that reason, it was not surprising to us that loss of Atg14, an essential autophagy protein, restored PBs in control cells. If PB turnover in all cells relies on autophagic flux, when it is blocked, PBs increase. Moreover, we have regraphed these data to show PB counts from each cell in the experiment, and determined the data to be statistically significant ($p<0.001$).

Observing the graphs in the figures, it seems that for some experiments, the authors used a different number of biological replicates (i.e. N=3, or N=4 or N>4); it would be informative if the authors could comment about those differences in sample size; e.g., outliers exclusion or technical difficulties.

Many of the experiments presented in this manuscript are extremely technically challenging. We use primary endothelial cells throughout the paper, and our dedication to use of this most biologically relevant cell type necessitates that overexpression is achieved by lentiviral transduction. Therefore, many of our experiments required sequential transduction of primary cells with i) a shRNA-expressing population of lentiviruses and ii) a KapB-expressing or control population of lentiviruses. Due to the technical challenges of these experiments, and the stress that dual transduction and selection places on primary cells, it is important to repeat some of these experiments three (minimum) or more times. For each independent experiment, a different

passage of HUVECS and a separate transduction is used. We have not used any stable cell lines in this paper. Moreover, we have now re-graphed all the data for PB quantification to increase transparency of experimental variability.

Figure 5: in line 290, the authors refer to Figure 5E, but there is no panel E in the figure. Moreover, there is no mention of panel D in the results section regarding figure 5.

We thank the reviewer for pointing out this oversight, and we have amended the manuscript to refer to both Fig 5D and Fig 5E. Panel 5E includes the quantification of representative images shown in panel 5D from three independent experiments. In this experiment, we examined what happened to PBs in the context of KapB expression, when endogenous NDP52 was silenced using shRNAs that targeted the 3' UTR and then NDP52 was overexpressed to rescue KapB-mediated PB disassembly. This is an extremely important part of Figure 5, as it solidifies that without NDP52, KapB-mediated PB disassembly is impaired.

Reviewer #2:

KSHV Kaposin B is shown to inhibit P-body (PB) formation by increasing autophagy. They show that KapB induced authophagy in a mechanism distinct from mTOR signaling, but dependent on ATG5 and ATG14. KapB is shown to induce ARE-containing cytokine mRNA expression. KapB and Torin are found to cause loss of Dcp1a scaffolding component of PBs in an autophagy dependent manner. They show that the NDP53 selective autophagy receptor is required for KapB dissolution of PBs. In contrast, MK2EE induction of PBs does not require Atg5. They also show that KSHV infection causes loss of PBs in a Atg and NDP53 dependent manner.

Overall, this is a technically strong paper with an important advance in understanding the role of KapB in regulating autophagic flux and how this further effects PBs and ARE-dependent mRNA expression. A major limitation of the paper is a lack of biochemical mechanism as to how KapB regulates autophagic flux. This is not addressed in this work.

No Major Concerns except the lack of any biochemical mechanism to account for KapB activities.

We thank Reviewer 2 for their positive feedback and appreciation of the importance of our study. To help the reviewer appreciate the data in this manuscript and how they build on former work, we have responded to this overall concern below.

Previous work showed that KapB activated the kinase MK2 (McCormick and Ganem, 2005), and that silencing or inhibition of MK2 diminished KapB-associated PB disassembly (Corcoran et al. 2015). That same year, active MK2 was implicated as a novel autophagy inducer that phosphorylated the master autophagy regulator, Beclin, at a non-canonical regulatory site, Ser90 (Wei et al. 2015). In the first submitted version of the manuscript, we showed that Beclin-1 was phosphorylated at Ser90 in response to KapB expression (Fig 2E of the revised manuscript), identifying the mechanism used by KapB to enhance autophagy. In the revised version of our manuscript, we add significantly to this analysis by adding the following pieces of data:

In Fig 2D, we show additional data that KapB expression does not influence ULK phosphorylation, further supporting that the mechanism of autophagy induction by KapB is independent of mTOR/ULK regulation, the canonical autophagy induction pathway. These data, combined with our previous work, provide a compelling mechanism for how KapB expression enhances autophagic flux, as follows: KapB binds and activates MK2; when active, MK2 phosphorylates Beclin at Ser90 to upregulate autophagic flux and induce PB turnover.

Moreover, we performed additional experiments and now show that primary KSHV infection of HUVECs enhanced autophagic flux (as determined by enhanced LC3 puncta formation) and increased phosphorylation of Ser90 of Beclin; these data are found in Fig 6A-B of the revised manuscript. Finally, to solidify a role for KapB in this mechanism, we used a recombinant KSHV that fails to express KapB (delB BAC16) and matched wild-type control (BAC16 KSHV) whose creation is described in our recent publication (Kleer et al. 2021). Primary infection of HUVECs with BAC16 KSHV also induced LC3 puncta formation and phosphorylation of Ser90 of Beclin, whereas infection with delB BAC16 virus failed to enhance autophagic flux or phosphorylation of Beclin-1 at Ser90. These data are presented in Fig 6C-D of the revised manuscript. We previously showed that infection with delB BAC16 failed to induce PB disassembly and enhance cytokine mRNAs in Fig 9 of (Kleer et al. 2021). Taken together, our new data significantly enhance the mechanistic analysis of KapB-induced autophagy in the context of KSHV infection and solidify the important role that KapB plays in mediating NDP52 autophagy-dependent PB disassembly during KSHV infection.

Minor
Fig 2D Western blot should be quantified. It is not apparent that there is a relative increase in phosphorylation.

We thank the reviewer for this observation. We have quantified the extent of Beclin Ser90 phosphorylation in response to KapB expression and these data are now presented in Fig 2E of the revised manuscript.

Fig. 3. Does "conditioned" media from KSHV infected SLK cells contain KSHV virus? What is the stimulating activity in this conditioned media?

PBs control cytokine transcript levels post-transcriptionally because they modulate mRNA decay; however, in untreated HUVECs, the transcription of these mRNAs is minimal. To detect changes in these transcripts by RT-qPCR, we need to activate their transcription. To activate cytokine transcription in the most biologically relevant way for KSHV infection, we chose to use culture supernatant from latently infected cells, as this culture supernatant would be expected to contain inflammatory stimuli e.g. TNF. The supernatant was derived from r219.KSHV latently infected iSLK cells; because latent cells do not produce viral progeny, the cell supernatant should not contain virus particles but should contain cytokines released in response to KSHV latent gene expression (including KapB). Nonetheless, to ensure there was no virus, we filtered the cell supernatant through a 0.22 μm membrane, as described in the methods. In short, KSHV latent cells produce inflammatory molecules, creating a feedforward loop to activate more inflammatory cytokine transcription and cytokine RNA stabilization.

Fig 4B. KapB reduction of Dcp1a is an important finding. The Western showing the decrease expression should be on the same blot (unspliced lanes if possible) similar to what is shown with Torin (Fig. 4A).

We thank the reviewer for their concern. We have replaced the western blot in Fig 4A with a new blot where all the samples are beside one another, removing the need to splice lanes.

Reviewer #3:

Robinson et al. address the connection between autophagy and inflammatory cytokine expression in KSHV Kaposin B (KapB)-expressing cells. The authors had previously demonstrated that processing bodies (PB) that regulate the stability of messenger RNAs encoding inflammatory molecules disassembled in response to KapB expression. This led to increased expression of inflammatory proteins. They now show that disassembly of PBs in KapB-expressing cells depends on the autophagy proteins Atg5 and Atg14 and the selective autophagy receptor NDP52. They also assert that KSHV infection causes similar disassembly of PBs via Atg5 and NDP52. While using autophagy to activate the inflammatory response is an intriguing mechanism for a virus that causes inflammation-associated diseases such as KS, there are several issues with the experiments and their interpretation that are described below.

Major criticisms:
1. Nearly all experiments (except Fig.7) were performed using cells expressing a protein of interest such as KapB. While KapB may indeed activate autophagy of PBs via the canonical autophagy pathway to elicit an inflammatory response, whether this actually happens in KSHV-infected cells is not clear. When expressed in isolation in un-infected cells, KapB may be capable of activities that may not be relevant in the context of infection. Instead of using KapB overexpression (in uninfected cells) as a surrogate for KSHV infection, these experiments need to be performed in infected cells with/without depletion of virus-expressed KapB. The study in its current form lacks virologic relevance.

We would like to thank Reviewer #3 for their insightful comments. We agree with this criticism of our work and have added several new pieces of data to the manuscript that highlight the virologic relevance of KapB-mediated autophagic increase to KSHV infection.

Autophagy regulation by KSHV is complex, as the virus encodes five gene products, at minimum, that have been shown to manipulate autophagic machinery. We therefore initiated our study using a reductionist approach wherein we transduced HUVECs with lentiviruses that express KapB, a system that uses biologically relevant primary cells and physiologically relevant levels of transgene expression. We have now completed a set of experiments showing that KSHV primary infection of HUVECs enhanced autophagic flux, despite the known expression of the negative autophagy regulator vFLIP during this replication phase (Fig 6A). To our knowledge, this is the first time that autophagic flux has been examined in primary endothelium during KSHV latency, although others have shown autophagic flux is elevated in latently infected B cells. To address the important role of KapB in autophagic upregulation after KSHV infection, we performed a study to compare autophagic flux after WT versus delB virus infection

of HUVECs. Using the delB BAC16 recombinant virus that failed to induce PB disassembly in HUVECs (Kleer et al. 2021, see Fig 9), we examined autophagic flux during KSHV infection by staining for LC3 puncta formation. These data show that delB BAC16 KSHV fails to increase LC3 puncta compared to BAC16 wildtype KSHV, indicating that KapB is necessary for upregulated autophagy during KSHV infection of primary endothelial cells (Fig 6C of revised manuscript). We also present data herein that show that limiting autophagic flux diminishes PB disassembly after KSHV infection (Fig 7). Taken together, these data suggest a model where KapB is the viral gene product responsible for enhancing autophagic turnover and mediating PB disassembly after primary infection of HUVECs.

To address the biochemical mechanism of autophagy upregulation by KapB during KSHV latency, we now show data indicating enhanced levels of Ser90 phosphorylation of Beclin 1 after KSHV infection of HUVECs (Fig 6B of the revised manuscript). These data show that KSHV induces phosphorylation of Ser90 while infection with delB virus does not (Fig 6D), consistent with our previous data that showed that ectopic expression of KapB induced the phosphorylation of Ser90 on Beclin 1 (Fig 2E). These data solidify the mechanism by which KapB upregulates autophagic flux during KSHV infection of HUVECs.

2. *There are issues with overinterpretation of data. For example, while there was a trend towards more PB when Atg5 and NDP52 were depleted, Fig.7 fails to show a statistically significant increase in PBs after Atg5 and NDP52 depletion and yet, the authors conclude that these proteins are required for KSHV-mediated PB disassembly. There also appears to be an error in p value calculation for the 6-hour IL-1b bar graphs in Fig.3A. The vector versus KapB bars do not appear to be significantly different and yet, there are two asterisks denoting high significance.*

We understand the reviewer's comment and apologize for the confusion. If provided with the opportunity to resubmit, we will ensure that the explanation of these data is clearer in the revised manuscript. The data, shown in Fig 7B of the revised manuscript, demonstrate the following: 1) KSHV *de novo* infection of HUVECs induced PB disassembly in control cells (non-targeting shRNA) compared to matched uninfected controls. 2) In cells where Atg5 or NDP52 are silenced, KSHV infection failed to induce significant PB disassembly compared to matched uninfected controls (Atg5 or NDP52 hairpins). The reviewer has expressed concern that when Atg5 or NDP52 are silenced and KSHV-infected cells are compared to control infected cells, there is not a significant increase in PBs. However, this is not the proper comparison to make. Silencing of NDP52 alters the baseline level of PBs; for this reason, we presented the data to demonstrate significant PB disassembly in the absence, but not in the presence, of silencing Atg5 and NDP52. We have added an additional explanation of these experiments to the revised manuscript (Lines 592-600). Moreover, we have re-graphed all the data for PB quantification to include individual PB counts/cell for every cell that was imaged for each condition and for each independent experiment. These new graphs provide greater data transparency which we are confident will assuage the reviewer's concerns; moreover, including all PB counts increased the statistical significance of our data in all experiments.

3. *The purpose of adding culture supernatant from iSLK cells in Fig.3A is unclear. This 'mimic'*

of the KS microenvironment has no discernible effect on KapB mediated effects on the abundance of cytokine transcripts.

We take full responsibility for the reviewer's confusion on this point, as we realize that the significance of this method was not highlighted in our manuscript. We have added the following information to the revised version of the manuscript (Lines 400-408) to clarify the utility of this approach.

To understand if PB disassembly correlated with changes to PB-regulated inflammatory cytokine transcripts, we performed RT-qPCR for the AU-rich containing mRNAs, IL-6, CXCL8, COX-2 and IL-1β (Fig 3A). PBs control cytokine transcript levels post-transcriptionally because they modulate mRNA decay; however, in untreated HUVECs, the transcription of these mRNAs is minimal. To detect changes in these transcripts by RT-qPCR, we need to activate their transcription. Rather than use a common inflammatory molecule to activate cytokine transcription e.g. lipopolysaccharide, we chose to activate cytokine transcription in the most biologically relevant way for KSHV infection. Infection with KSHV induces high levels of pro-inflammatory cytokines, chemokines and angiogenic factors such as TNF, IL-1α, IL-1β, IL-6, CXCL8, IFN-γ, VEGF, COX-2, and GM-CSF. Therefore, we used culture supernatant from latently infected cells, as this culture supernatant would be expected to contain inflammatory stimuli. The supernatant was derived from latently infected iSLK cells; because latent cells do not produce viral progeny, the cell supernatant should not contain virus particles but should contain cytokines released in response to KSHV latent gene expression (including KapB). Nonetheless, to ensure there was no virus, we filtered the cell supernatant through a 0.22 μM membrane, as denoted in the methods. In short, KSHV latent cells produce inflammatory molecules, creating a feedforward loop to activate more inflammatory cytokine transcription and cytokine RNA stabilization.

4. *Overall, the data tying KapB to Dcp1a loss in Fig.4 is not convincing. In particular, autophagic loss of Dcp1a does not appear to be specific to the presence of KapB (Fig.4D). It seems that even at baseline, there is regulation of Dcp1a by autophagy. If anything, on the western blot shown in Fig.4D, Dcp1a recovery in the presence of shAtg5 is better when KapB is absent. Furthermore, loss of Dcp1a when KapB is overexpressed is subtle compared to when autophagy is induced by Torin (Fig.4B versus 4A). Also, KapB's effect on the SAR p62 appears to be more prominent than its effect on Dcp1a in Fig.4B. This is ignored by the authors.*

We thank the reviewer for these concerns, and we will address them each separately.

<u>Re: autophagic loss of the PB protein Dcp1a - is it specific to only KapB-expressing cells?</u>
We appreciate the reviewer's statement and agree with it. When autophagy is upregulated by KapB or by other inducers like Torin, steady-state level of Dcp1a protein is decreased. This suggests that the turnover of the protein is regulated by autophagy no matter what the inducer. This means that autophagy does not only regulate Dcp1a levels in KapB-expressing cells. By extension, since Dcp1a is important for PB stability, this suggests that autophagy is responsible for baseline turnover of PBs, analogous to its role in the baseline turnover of organelles like mitochondria. When KapB is expressed, or Torin is used to increase autophagic flux, the turnover of Dcp1a enhanced. We showed that KapB upregulated autophagy via the enhanced

phosphorylation of Beclin-1 at Ser90 (Fig 2E), thereby accelerating the turnover of PBs above baseline. This model is consistent with our data showing that KapB induces PB disassembly but does not block PB formation (Fig S1). Torin is a much more potent inducer of autophagy than KapB. Torin induces autophagy via activation of the ULK complex (see new Fig 2D), a canonical method of autophagy induction. KapB does not use this pathway to induce autophagy (Fig 2D). Thus, based on their different mechanisms of autophagy induction, it is not surprising that Torin treatment decreased steady-state Dcp1a protein level more than KapB. This feature of our data also suggests that elevated autophagy (by either mechanism) induces PB disassembly above the baseline level of granule turnover. This is also consistent with data that shows that silencing of Atg5, in the absence of KapB, enhances Dcp1a levels.

Re: reduced steady-state levels of the SAR, p62, in KapB-expressing cells
I fear that the reviewer has misinterpreted the significance of the p62 immunoblotting in this figure, and we apologize for the lack of clarity on this point. p62 is a selective autophagy receptor that delivers target cargo to the nascent autophagosome where it is degraded alongside the cargo. Therefore, if autophagic flux is elevated, by Torin or KapB, p62 turnover by autophagy would be expected to be elevated, and this should be reflected by a decrease in steady state p62 protein level. This is precisely what we have observed in Fig 4B. For this reason, p62 immunoblotting is often used to corroborate LC3-II immunoblotting as an indicator of autophagic flux. We have used immunoblotting of p62 as a reporter of enhanced autophagic flux. Our sincere apologies for the lack of clarity regarding this autophagy marker, additional statements have been added to Lines 223-233 and 458-460.

Minor
1. The description in the text for Figs.5D and E do not match what is shown in the panels or the legend. There is also reference to Fig.5F in the text which does not exist in Fig.5.

Thank you to the reviewer for pointing out our mistake. Apologies, the text has been modified in lines 494-495 to correct the erroneous reference to Fig 5F.

References

Corcoran JA, Johnston BP, McCormick C. Viral activation of MK2-hsp27-p115RhoGEF-RhoA signaling axis causes cytoskeletal rearrangements, p-body disruption and ARE-mRNA stabilization. PLoS Pathog. 2015;11: e1004597.

Kleer M, MacNeil G, Adam, N. Pringle ES, Corcoran JA. A panel of KSHV mutants in the polycistronic kaposin locus for precise analysis of individual protein products. J. Viol. 2021;doi: 10.1128/JVI.01560-21 Online ahead of print.

McCormick C, Ganem D. The Kaposin B Protein of KSHV Activates the p38/MK2 Pathway and Stabilizes Cytokine mRNAs. Science. 2005;307: 739–741.

Pringle ES, Robinson C-A, McCormick C. Kaposi's Sarcoma-Associated Herpesvirus Lytic Replication Interferes with mTORC1 Regulation of Autophagy and Viral Protein Synthesis. J Virol. 2019;93: e00854-19.

Wei Y, An Z, Zou Z, Sumpter R, Su M, Zang X, et al. The stress-responsive kinases MAPKAPK2/MAPKAPK3 activate starvation-induced autophagy through Beclin-1 phosphorylation.
