# Supplementary material for: Kaposi’s sarcoma-associated herpesvirus (KSHV) utilizes the NDP52/CALCOCO2 selective autophagy receptor to disassemble processing bodies
Source: PLoS Pathog. 2023 Jan 12;19(1):e1011080. doi: 10.1371/journal.ppat.1011080 (PMC9876383; doi:10.1371/journal.ppat.1011080)
Supplement: Attachment — Submitted filename: 2022-04-23 Robinson Singh Response to Reviewers Final.pdf [file ppat.1011080.s009.pdf]

Response to Reviewers
Robinson Singh *et al.*

Summary Comments

Reviewer #1: This is a revised version of the manuscript that focuses on KSHV KapB modulation of NDP52-mediated selective autophagy to target PBs for disassembly. In previous version, the key issue was the biological relevance of these findings to KSHV infection. In the revision, the authors added new results (Fig 6) to address this issue.
Overall, a lot of experiments have been performed in this project. The involvement of NDP52-mediated selective autophagy in KSHV latency is interesting and novel topic. Most experiments were well controlled and rigorously designed with valid rationale. The data showing that the autophagy plays a role in PB loss (as indicated by Dcp1a loss) is solid, as evidenced by different approaches, including Torin1 inhibition of mTOR, BafA1 inhibition of autophagy activity, and ATG5 depletion.

We thank the reviewer for acknowledging how much work has gone into this manuscript and its extensive improvement since the first submission.

Reviewer #2: The authors have responded point-by-point to the reviewers' comments. The use of recombinant KSHV viruses, including delB BAC16, makes the paper stronger. Nonetheless, there remain still some concerns to address.

We thank the reviewer for their appreciation of our detailed response to reviewers and the addition of significant data to the revised version that strengthen the paper. In the response that follows, we have endeavored to address additional concerns of this reviewer. However, we note that many concerns were not in reference to new data and refer to data that was deemed acceptable during the first round of review.

Reviewer #3: In this revised manuscript (PPATHOGENS-D-22-00182), the authors explored the interplay between KapB and processing bodies. They show that KapB directly influences Beclin phosphorylation leading to autophagic pathways activation and further PB disassembly. The authors have a long history of studying this process, and in this article they provide important new details on the mechanism underlying KapB-mediated PB disassembly. This is a technically challenging mechanism to study and a complex multiplayer story. Yet, the authors do an elegant balancing act of explaining their results while providing sufficient details to support their claims. Based on the previous round of reviews, there were concerns about physiological relevance of the findings and the authors have greatly added to their original manuscript to address these concerns. In particular, they have generated a KapB deletant KSHV backbone to test directly the effect of KapB on this cascade and have infected primary cells to show that the observed effect on autophagic flux was KapB-dependent. The findings are important and of interest for the KSHV community.

We sincerely thank Reviewer 3 for their appreciation of the significance of our work and their acknowledgement that the new additions greatly improved our original submission and addressed all major concerns from the prior review.

Major Comments

Reviewer #1:

1. The biological relevance issue has been addressed with de novo KSHV infection assays. The use of a KapB-deficient KSHV is a powerful tool in this regard (Fig 6, C-D). However, the KapB-deficient KSHV was only used for primary infection, instead of latent infection (which was done for KSHV.219, Fig 6A). This project focuses on KSHV latency, and it is notable that autophagy plays different or even opposite roles in primary vs latent viral infection. Furthermore, the outcomes were only assessed by confocal microscopy for LC puncta deregulation, which is not convincing. More important evaluations including immunoblotting for PB loss (Dcp1a as a marker) and autophagy involvement should be done to consolidate the conclusion and improve the consistency of data throughout the project.

We thank the reviewer for the acknowledgement of the significance of our new data. In response to the reviewer's major concerns, we have performed additional experiments and added new data to the revised version of the manuscript.

In Fig 6A of our original manuscript (Fig 7A of the revised version), we performed primary infection of HUVECs with KSHV.219 and permitted latency establishment, after which an analysis of LC3 puncta was performed. In Fig 6C of our original manuscript (Fig 7C of the revised version), we likewise performed primary infection of HUVECs with WT or delKapB BAC16 viruses and after latency establishment, analysis of LC3 puncta was performed. Although the conditions of infection used in both cases support KSHV latency establishment (see our use of the same infection model in Kleer et al. 2022), to address the reviewer's concern, we infected iSLK cells, a KSHV producer cell line known to exhibit tight control over latency (Myoung and Ganem 2011). Seven days after infection, we compared autophagic flux between WT BAC16 or delKapB BAC16 latent iSLK cells by immunoblotting for the lipidated form of LC3 (LC3-II) prior to and after the treatment of each sample with Bafilomycin A1 (BafA1), an inhibitor of autophagosome-lysosome fusion that prevents LC3-II (and other cargo) from degradation. The difference in LC3-II level before versus after BafA1 treatment serves as a measure of autophagic flux in each condition. These new data are presented in Fig 7E of the revised paper and reveal that although autophagic flux is high in WT BAC16 cells, it is significantly diminished in delKapB BAC16 latent iSLK cells.

In response to the reviewer, we also performed immunoblotting for Dcp1a after infection with the KSHV.219 virus and demonstrated that KSHV infection resulted in a significant decrease in the steady state level of Dcp1a protein. These data are found in Fig 8E of the revised paper.

2. Another important experiment to consolidate the biological relevance is to deplete KapB (by shRNA or sgRNA) in KSHV HUVECs, followed by evaluation of relevant outcomes and phenotypes. I appreciate they authors did loss-of-function assays using shRNA-mediated knockdown for NDP52 and ATG5, but similar experiments for KapB are more important in terms of biological relevance.

We thank the reviewer for this suggestion, but respectfully suggest that our use of recombinant virus technology as a method to eliminate KapB is a far superior approach to shRNA-mediated RNA silencing. In our first study on KapB-mediated PB disassembly, we utilized RNA silencing to eliminate the kaposin transcript (Corcoran et al. 2015, PLOS Path). These experiments revealed that knockdown of the kaposin RNA eliminated KSHV-mediated PB disassembly in latent endothelial cells. KapB and two other *kaposin* polypeptides are translated from overlapping ORFs encoded by this polycistronic RNA transcript. RNA silencing is an RNA-targeting method that eliminates the entire transcript and impacts the production of all three kaposin proteins, making this strategy non-specific to KapB. In response to presentation of this work at conferences, several KSHV researchers commented on the importance of building a KSHV recombinant virus that specifically lacked KapB to show the specificity of the phenotype.

In response to this feedback, we dedicated much time and effort to build a delKapB recombinant KSHV virus, capitalizing on the degeneracy of the amino acid code to recode the KapC reading frame (which overlaps that of KapB) such that we retained all the amino acids in KapC while simultaneously destroying the amino acid sequence in the KapB frame. Building this recombinant virus was a significant undertaking which was described in detail in our recent publication (Kleer et al. 2022). Using the delKapB BAC16 virus, we confirmed that KapB was not only sufficient, but necessary, for KSHV-mediated PB disassembly (Kleer et al. 2022); herein we use the same virus to confirm that KapB is not only sufficient, but necessary, for KSHV-mediated enhancement in autophagic flux. This recombinant virus is the superior approach to answer our question about the specific requirement for KapB in the KSHV-mediated autophagy increase.

3. It would be also important to check if KSHV latent infection induces NDP52 expression, considering the fact that EBV induces the selective autophagy receptor p62 in its latency and employs p62-mediated selective autophagy for DNA damage response.

We thank the reviewer for this suggestion. We infected HUVECs with KSHV.219 virus and did not observe an increase in NDP52 levels after infection (Fig 8C of the revised manuscript). Likewise, we did not observe NDP52 increases after KapB expression (Fig S4C, S6A). This was somewhat surprising to us as others have shown that KSHV infection increases the activity of the Nrf2 transcription factor and NDP52 is an Nrf2-responsive gene (Gjyshi et al. 2015).

4. The link of MK2 to KapB-elicited autophagy is missing. That is said, the authors' hypothesis that KapB induces autophagy through a MK2-dependent manner was not tested in the manuscript, although the authors have shown in their previous papers that KapB interacts with MK2, and MK2 can phosphorylate Beclin1. A loss-of-function assay of MK2 or an assay with a KapB MK2-interacting deficient mutant is needed to answer this question.

Our previous work utilized shRNA-expressing recombinant retroviruses to silence MK2 and showed that knockdown of MK2 decreased KapB-mediated PB disassembly (Corcoran et al. 2015). These data indicated that MK2 played an important role in the mechanism of PB disassembly induced by KapB. Since that time, we entirely replaced the recombinant retrovirus system in our lab with a second generation recombinant lentivirus system to improve transduction efficiency of HUVECs. We attempted to create new shRNA-expressing lentiviruses to silence MK2 and examine if KapB expression was able to induce autophagic flux (since our prior work already determined the role of MK2 in PB disassembly). However, in the time allocated to complete this review, our attempts to silence MK2 in HUVECs were unsuccessful, as shown here. Because HUVECs are primary cells, we have previously observed that not all shRNAs work consistently in all batches of HUVECs due to donor variability. Despite our inability to silence MK2 in this study, the work in this manuscript adds significantly to our understanding of the role of MK2 in the induction of autophagy by KapB. We have shown that KapB and KSHV latent infection elevate autophagic flux via a mechanism that requires phosphorylation of serine 90 of Beclin, and others have shown that this the same non-canonical mechanism of autophagy induction is used by MK2. Like the constitutively active form of MK2, KapB failed to activate autophagic flux by canonical pathways that are used by Torin. To our knowledge, only two serine/threonine protein kinases have been shown to phosphorylate Beclin at serine 90, MK2 and calcium/calmodulin-dependent protein kinase II (CaMKII) (Wei et al. 2015, Li et al. 2017), making this an atypical autophagy initiation mechanism. These data are presented in Fig 2 of the previous version of our paper (Fig 3 of this revision).

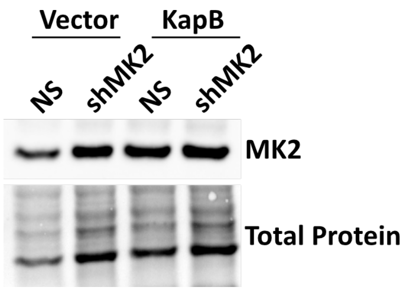

Fig 1. Failure to silence MK2 in HUVECs using shRNA-expressing lentiviruses.

Reviewer #2:

1. While the authors provided the biochemical data on how KapB induces autophagy flux, how KapB activates selectively NDP52-mediated autophagy is not clear yet. Fig. 7C depicts the direct interaction between Dcp1a (or other PB protein) and NDP52, but the manuscript does not show any biochemical evidence to support their interactions.

We thank the reviewer for the suggestion. We now provide additional biochemical evidence to support our model. In response to the reviewer's comment, we overexpressed tagged NDP52 and tagged Dcp1a and performed a co-immunoprecipitation experiment. Tagged NDP52 was immunoprecipitated, and under these conditions, it failed to co-immunoprecipitate tagged Dcp1a. We then repeated this experiment using tagged NDP52 and a tagged version of the PB-resident protein, Pat1b. We observed that immunoprecipitation of NDP52 did co-immunoprecipitate Pat1b, providing the molecular basis for an interaction between the selective autophagy receptor NDP52 and a PB protein. These data have been added to Fig 8D in the revised manuscript. Pat1b is a decapping cofactor that has been shown by others to interact with several PB-localized proteins, including DDX6, Dcp1a, and Hedls. Due to its ability to interact with many PB components, it is considered a fundamental scaffolding protein for PBs (Vindry 2019). Although Pat1b is not essential for PB formation, its loss has previously been shown to promote PB

disassembly and reduce PB foci. This fits well with our other observations, as an interaction between NDP52 and Pat1b could also serve to deliver Dcp1a to the autophagosome for degradation, consistent with our observations of Dcp1a protein decrease.

After we obtained this co-immunoprecipitation result, we performed additional immunoblots after KSHV infection or Torin treatment to determine if there was an impact on the steady-state levels of endogenous Pat1b. We speculated that if Pat1b was found in a complex with NDP52, its steady-state levels would decrease in response to these stimuli. We observed that KSHV infection resulted in a significant decrease in steady-state Pat1b by immunoblotting, new data that is now found in Fig 8E. Torin treatment also showed a significant decrease in steady state Pat1b and Torin treatment in the context of NDP52 knockdown restored Pat1b protein to control levels. We note that these analyses capture the total Pat1b in the cell population, not only PB-localized Pat1b. Endogenous Pat1b resides in several subcellular locations including the cytoplasm, the nucleus, Cajal bodies and PBs. This diverse localization makes it difficult to observe changes to total Pat1b levels with a treatment that would be expected to only impact PB resident Pat1b pools (Vindry 2019). Despite this, we were able to observe a decrease in steady-state Pat1b levels. These data are shown in Fig 6C of the revised manuscript.

The results of these co-immunoprecipitation and immunoblotting experiments have now been incorporated into our model and this figure has been updated. The revised model is found in Fig 8F of the revised manuscript.

2. The other SAR Calcoco1 and Calcoco3/TAX1BP1 are paralogs that most resemble NDP52. Thus, it'd be nice to comparatively examine the activities of Calcoco1 and Calcoco3/TAX1BP1 in PB dismantling, Dcp1a interaction, and ARE-mRNA reporter expression along with NDP52.

NDP52 is a member of the Calcium-binding and coiled-coil domain containing (CALCOCO) family and shares high sequence homology and domain organization with other members Calcoco1/CoCoA and Calcoco3/TAX1BP1. Although TAX1BP1 has also been shown to act as a selective autophagy receptor, CoCoA has traditionally been viewed as transcription coactivator. Neither protein has been implicated to the extent as NDP52 in selective autophagy; however, we agree with the reviewer that these activities would be interesting to investigate in future studies.

3. The authors determined the relative intensities of immunoblotting (IB) bands only by normalizing to that of control band. However, some IB samples were not equally loaded cross the lanes, shown by the levels of total proteins. Equal loading of the samples, particularly in Figs. 1A, 2E, 4B, 4D, 7B (shAtg5), S2A, S2B, S3A, S3B, and S5A, may not support the conclusions drawn in this paper.

For all quantification of immunoblot bands presented in our manuscript, we performed the following analyses. First, we used densitometry to quantify total protein loaded in each lane. Second, we measured the band of interest e.g. p62 in matched control cells. Third, we measured the band of interest e.g. p62 in the experiment, such as in KapB-expressing cells. Finally, we performed a two-step normalization, wherein the densitometry result was first normalized to total protein for that lane and then to the matched control band. The following statement is found in every applicable figure legend, "*Samples were quantified by normalizing the [insert] protein*

levels to the total protein in each lane and then the [insert] control using ImageLab (Biorad)”. However, to make this clearer for the reader and the reviewer, we have altered the y-axis label for every graph in the manuscript that measures band intensity to note this dual normalization step.

Minor Comments

Reviewer #1:

1. Since the central finding of this study is that NDP52-mediated selective autophagy is involved in PB disassembly in KSHV latency, one outstanding question is about the definition of NDP52-mediated selective autophagy. In terms of p62-mediated selective autophagy, it requires substantial post-translational modification on p62, i.e. site-specific phosphorylation and ubiquitination, which are required for target recognition and transportation. The authors should evaluate site-specific phosphorylation (and/or ubiquitination) of NDP52, which is likely required for its function in PB clearance, in the context.

We thank the reviewer for this thoughtful suggestion. Post-translational modification of PB proteins and/or NDP52 is indeed the next logical step in our study; however, this manuscript contains 8 main figures and 6 supplemental figures, making this work beyond the scope of the current publication.

2. Fig 1A. It seems like the basal autophagy activity in HUVECs is very high, compared 0 h between Vector vs KapB samples. LC3-II should be at very low level if the basal autophagy activity is low. The high basal level of autophagy activity in HUVECs is further shown by low level of p62 and PB components such as Dcp1a (Fig 4, B, D). Thus, further induction of autophagy by overexpressed KSHV KapB in these cells may not be significant. This is particularly true considering the marginal difference of p62 downregulation (the endogenous level of p62 is already very low due to high endogenous autophagy activity) in the Vector vs KapB group in immunoblotting results (Fig 1A).

We thank the reviewer for the insightful comments on our data. We apologize but we are unclear of the particular question or request being posed by the reviewer at this time.

If basal autophagy is low, there should be equal levels of LC3-I and LC3-II in the cell. Treatment with BafA1 blocks the final degradative step of autophagy, and it is a requirement for the study of the dynamic flux of autophagy in the system. A comparison of LC3-II level between different treatments/cells at basal conditions is not accurate because the steady state level of this protein varies with the cell cycle and other inputs. To accurately compare autophagic flux between different conditions requires the concomitant assessment of LC3 conversion (how much LC3-I is lipidated to form LC3-II) and turnover (how much LC3-II accumulates when its degradation is blocked versus how much LC3-II is present when degradation is permitted). Please see the guidelines for the study of autophagy (Klionsky 2021).

3. Fig 4D. The results are not convincing and the current experimental design for disclosing role of Atg5 is not correct. The current design is to disclose KapB role in ATG5-mediated

upregulation of Dcp1a. On the figure, I do not see KapB causes a loss of Dcp1a (lanes 1 vs 3), nor Atg5 depletion restores any loss of Dcp1a (lanes 2 vs 4). The statistical analysis on lanes 3 vs 4 is not correct since this comparison cannot disclose a role for Atg5 in this process.

We apologize for the confusion caused by our analyses and will endeavor to explain these data more clearly here. The data shown in Fig 4D of the prior submitted manuscript (Fig 5D of the revised version) have been quantified and represent three independent repetitions of the experiment. Only one representative blot is shown. As the quantification denotes, in NS-treated cells, there is a significant reduction in the steady-state level of Dcp1a after KapB expression (lanes 1 and 3). To ask if knockdown of Atg5 restores Dcp1a protein in the context of KapB expression, we compared Dcp1a protein level between KapB-expressing NS-treated cells and KapB-expressing shAtg5-treated cells (lanes 3 and 4) and found a significant restoration of Dcp1a protein in three independent experiments. We have altered the axis of this graph to more accurately reflect how the densiometric quantification was performed.

4. Evidence on Dcp1a loss caused by autophagy induction (e.g. Torin1 inhibition) is solid. However, Recapitulation with KapB on Dcp1a loss is not very convincing. For example, Fig 4B. The IB blot on KapB downregulation of steady-state Dcp1a (also p62) is not convincing; no difference between lanes 1 vs 3 on the blot. The authors should show the best IB result from the triple independent experiments.

The data shown in Fig 4B of the prior submitted manuscript (Fig 5B of the revised version) represent three independent experiments. We have added a different representative blot to Fig 5B that more accurately reflects the densiometric analyses of these experiments. Moreover, we have also added new data to the revised version of the manuscript that shows that the PB proteins Dcp1a and Pat1b both decrease after KSHV infection (Fig 8E of the revised version), a result that is consistent with the data presented in Fig 5B.

5. Fig 2. Page 12. To my knowledge, ULK1 S555 is targeted by AMPK for phosphorylation, not a target of mTOR.

Active mTOR phosphorylates Atg13 and Ulk1/2, suppressing Ulk1/2 kinase activity and limiting autophagy. Under starvation conditions or when mTORC1 activity is pharmacologically inhibited by Torin, these sites are rapidly dephosphorylated (Alers 2012). We apologize for the erroneous wording in the relevant sentence of our results section (page 12). This sentence has been re-worded to eliminate the confusion.

6. Fig 5. Immunoblotting is needed to evaluate Dcp1a recovery of Torin1-induced loss in NDP52-depleted HUVECs, although different approaches, including loss-of-function and rescue assays were used. Immunoblotting on Dcp1a regulation would make the data consistent in the study and consolidate the conclusion.

We thank the reviewer for this helpful suggestion. After performing co-immunoprecipitation using tagged NDP52, we discovered that NDP52 did not co-immunoprecipitate Dcp1a but did co-immunoprecipitate Pat1b, a PB protein that plays an important scaffolding role and interacts with Dcp1a. Therefore, we performed immunoblotting for both Dcp1a and Pat1b after Torin

treatment, with and without NDP52 silencing. We observed significant decrease in the steady state levels of both proteins after Torin treatment. In the context of NDP52 knockdown, Pat1b protein level in Torin treated cells was restored to control levels; however, Dcp1a protein level was not fully restored after NDP52 knockdown. These data are now shown in Fig 6C of the revised manuscript.

7. It is an interesting question if NDP52-mediated autophagy for PB clearance also happens in KSHV PEL cells. This study only used HUVECs. This can be discussed.

We thank the reviewer for this point. We did not analyze autophagic flux in KSHV-infected B-cells but would refer the reviewer to a recent study that demonstrated that latent BCBL-1 cells also displayed enhanced autophagic flux (Pringle 2019). In the discussion, we note that autophagy is also elevated in latent BCBL-1 cells, suggesting the possible application of this mechanism to KSHV-induced B-cell lymphomas.

8. Fig2B. Statistical analysis is missing

We apologize for the oversight and thank the reviewer for pointing it out. The statistical analysis of this experiment has been added to Fig 3B (formerly Fig 2B) of the revised manuscript.

Reviewer #2:

4. Like p62 in Fig. 1A, is it expected that the level of NDP52 is decreased by KapB but restored by Baf A1 treatment?

We thank the reviewer for this question. Neither KapB expression nor KSHV infection altered NDP52 levels. Although NDP52 plays an important role as a SAR, it has many alternative roles, and it is not typically used as a measure of autophagic flux.

5. The data of the NDP52 complementation experiments in Figs. 5D/5E and S6B/S6C seem controversial. In Figs. S6B and S6C, why could not NDP52 complementation in shNDP52 control cells induce PB disassembly? referring to vector controls

The reviewer is correct in their interpretation. When NDP52 is added back to NDP52-silenced control cells, PB disassembly is not induced. In this case, elevated levels of NDP52 are not sufficient to induce elevated autophagic degradation of PB components. KapB expression, KSHV infection, or Torin treatment are needed to elevate autophagic flux.

6. In Fig. 3A, does KapB expression significantly enhance the expression of IL-6 at 0 h? If not, like Torin (Fig. 3B), is it expected that the iSLK 219 culture media can promote IL-6 expression in the presence of Torin? Otherwise, it'd be better to remove the results of the experiments using the conditional media in Fig. 3A. It is not clear if the media promoted the expression of the target genes tested, as not provided the statistical significance between 0 and 6 h in either vector or KapB cells.

Inflammatory cytokines are highly regulated molecules. Their translation into protein is regulated at two distinct stages: transcriptional initiation and post-transcriptional decay and/or translational suppression in PBs. Because of the potent biological implications of producing high levels of these cytokines this post-transcriptional regulation is important and it is mediated via 3' AU-rich elements in the 3' UTRs of many cytokine transcripts that direct them to PBs. Therefore, transcriptional induction will increase these RNA levels only modestly unless also accompanied by abrogation of post-transcriptional decay/suppression in PBs. We did not expect to observe elevated levels of ARE-containing cytokine RNAs without activating both transcription and RNA stabilization mechanisms. In Fig 4A (formerly Fig 3A), we used untreated HUVECs where the transcription of most ARE-mRNAs is minimal. To activate transcription of ARE-containing cytokines in a biologically relevant manner, rather than using common inflammatory stimuli such as the bacterial product, lipopolysaccharide, we treated KapB-expressing or control HUVECs with 0.22 μm-filtered conditioned media from KSHV latently infected cells. Conditioned media from latent KSHV cells would be expected to contain inflammatory cytokines, e.g. TNF, that are elevated in KS, without containing virions. Although we expected conditional media to enhance inflammatory cytokine transcription, given the rapid turnover that most of these cytokine transcripts exhibit, we also expected that KapB expression would further enhance RNA levels post-transcriptionally. Consistent with this dual regulation, KapB alone (no transcriptional stimulus) did not significantly enhance cytokine RNA levels except for IL-1β, but KapB expression plus transcriptional stimulus significantly enhanced IL-6 and IL-1β levels. CXCL8 RNA levels were also elevated after both transcriptional activation and KapB, albeit not significantly, likely due to one outlying experiment. We respectfully disagree with the reviewer's comment in this regard, and suggest that our data from these experiments reflects the tight regulation exhibited by most ARE-containing cytokine RNAs.

If the authors anticipate a synergistic or additive role of the conditional media and KapB in the expression of ARE-containing cytokine genes, it is possible also to consider that proinflammatory cytokines induced in KapB-overexpressed cells or the context of latently infected (KapB+) cells may promote PB autophagy, i.e, via autocrine signaling in addition to KapB-mediated MK2 activation. This possibility may be examined using the conditional media generated from wild-type and delB BAC16 iSLK cells.

We thank the reviewer for this excellent suggestion. The possibility that latent infection (with KapB) may promote further PB autophagy via autocrine signaling in addition to cell autonomous effects is one that we have considered. This idea formed the basis for our use of conditioned media in Fig 3 (revised Fig 4) in the current study and is discussed above.

Reviewer #3:

1. Primary KSHV infection induces autophagy despite vFLIP: have the author check vFLIP expression in these cells?

We appreciate this comment and agree that vFLIP would be expected to diminish autophagic flux during latency. We tried to immunoblot for vFLIP to detect its expression using two different commercial antibodies, without success. However, we are confident that vFLIP is expressed in our system. In data recently published in Kleer et al. 2022, we determined that

KapB was not necessary for KSHV-induced cell spindling after primary infection and latency establishment in HUVECs. In these experiments, cells infected with the delKapB virus retained the ability to spindle in the absence of KapB. Since vFLIP is the only other KSHV latent gene produce reported to be sufficient for cell spindling, we reasoned that vFLIP was responsible for spindling primary endothelial cells when KapB was absent.

2. I understand that they author tried to mimic natural infection by infecting HUVECS but why not use cells where KSHV latency is established like BCBL or iSLKs where they could directly compare the effect to the lytic cycle?

We thank the reviewer for their suggestion. In our lab we attempt, when possible, to use the most relevant model for the biology of KSHV-induced Kaposi's sarcoma, namely infection or transduction of primary endothelial cells. iSLK cells are extremely useful producer cell line for making KSHV particles or BAC16 recombinant viruses; however, we have found that latent iSLK cell lines do not always recapitulate the biology we observe in primary endothelial cells. This may be because these cells are not of endothelial origin (Sturzl et al. 2013) or because the latent iSLK cells generated by us and others for the purposes of producing high numbers of virions require several weeks of hygromycin selection to obtain a population of cells that harbour viral genomes. Moreover, we quantified the genome copy number between our WT BAC16 and delB BAC16 iSLK cell lines and found that these cell lines contain drastically different amounts of the viral episome (as detailed in Kleer et al. 2022). For that reason, analysis of any virus-related biological phenotype would be inaccurate if performed in these latent producer cell lines. For these reasons, we performed *de novo* infection of naïve iSLKs with WT BAC16 and delB viruses. We waited one week to ensure tight latency establishment after which we analyzed these infected cells for autophagic flux using by quantifying the amount of baseline LC3-II versus LC3-II accumulation after BafA1 treatment for each infection. These data showed that delKapB-infected iSLK cells did not display enhanced autophagic flux as observed in WT BAC16-infected iSLK cells (Fig 7E).

We did not analyze autophagic flux in KSHV-infected B-cells but would refer the reviewer to a recent study that demonstrated that latent BCBL-1 cells also displayed enhanced autophagic flux (Pringle 2019).

3. Figure S1 is important – consider adding it as a main figure.

We thank the reviewer for the appreciation of these data. In response to this suggestion, we moved Fig S1C from the original submission into the main figure set, and it is now Fig 1C of the revised manuscript. This change made Fig 1 too unwieldly, and thus we moved the remaining panels from Fig 1 into Fig 2 of the revised manuscript.

4. No the authors' fault but the quality of the figures I received was bad (very pixelized) so please ensure that figure quality is checked

Apologies to the reviewer, we did upload high quality tiffs for every figure upon submission.

References

Alers…Stork. 2012. Role of AMPK-mTOR-Ulk1/2 in the Regulation of Autophagy: Cross Talk, Shortcuts, and Feedbacks. Mol. Biol. Cell. 1:2-11

Corcoran et al. 2015. Viral activation of MK2-hsp17-p115RhoGEF-RhoA signaling axis causes cytoskeletal rearrangements p-body disruption and ARE-mRNA stabilization. PLOS Path. 11(1):e1004597. doi: 10.1371/journal.ppat.1004597.

Gjyshi et al. 2015. Kaposi's sarcoma-associated herpesvirus induces Nrf2 activation in latently infected endothelial cells through SQSTM1 phosphorylation and interaction with polyubiquitinated Keap1. J. Virol. 89(4):2268-86. doi: 10.1128/JVI.02742-14.

Kleer et al. 2022. A Panel of Kaposi's Sarcoma-Associated Herpesvirus Mutants in the Polycistronic Kaposin Locus for Precise Analysis of Individual Protein Products. J Virol. 96(5):e0156021. doi: 10.1128/JVI.01560-21.

Klionsky et al. 2021. Guidelines for the use and interpretation of assays for monitoring autophagy (4th edition) Autophagy. 1:1-382. doi: 10.1080/15548627.2020.1797280.

Li et al. 2017. CaMKII-mediated Beclin 1 phosphorylation regulates autophagy that promotes degradation of Id and neuroblastoma cell differentiation. Nat. Comm. 8:1159.

Myoung and Ganem. 2011. Generation of a doxycycline-inducible KSHV producer cell line of endothelial origin: maintenance of tight latency with efficient reactivation upon induction. J Virol Methods 174(1-2):12-21. doi: 10.1016/j.jviromet.2011.03.012.

Sturzl et al. 2013. Kaposi's sarcoma-derived cell line SLK is not of endothelial origin but is a contaminant from a known renal carcinoma cell line. International Journal of Cancer. 132:1954–1958

Pringle et al. 2019. Kaposi's Sarcoma-Associated Herpesvirus Lytic Replication Interferes with mTORC1 Regulation of Autophagy and Viral Protein Synthesis. J Virol. 93(21):e00854-19. doi: 10.1128/JVI.00854-19.

Vindry, Weil, Standart. 2019. Pat1 RNA-binding proteins: multitasking shuttling proteins. RNA 10(6):e1557. doi: 10.1002/wrna.1557.
