# Supplementary material for: Kaposi’s sarcoma-associated herpesvirus (KSHV) utilizes the NDP52/CALCOCO2 selective autophagy receptor to disassemble processing bodies
Source: PLoS Pathog. 2023 Jan 12;19(1):e1011080. doi: 10.1371/journal.ppat.1011080 (PMC9876383; doi:10.1371/journal.ppat.1011080)
Supplement: Attachment — Submitted filename: 2022-11-06 Response to Reviewers FINAL.pdf [file ppat.1011080.s010.pdf]

Response to Reviewers' Concerns
Major Issues

Reviewer #1

1. The involvement of MK2 in KapB-induced autophagy. The proposed pathway "KapB-->MK2-->Becline1 phosphorylation-->autophagy" is not complete since evidence for the involvement of MK2 is not provided. As Reviewer 2 also pointed out in previous version, "how KapB activates selectively NDP52-mediated autophagy is not clear yet". The authors argued that they had difficulties in silencing MK2 in HUVECs. However, they did silence ATG4 and ATG14 (Fig 2), and ATG5 and NDP52 (Fig 8) in these cells. So there is no technical challenges. Replacement of different shRNAs should address this issue.

In the newly revised version of our manuscript, we provide new data (Figure 8) to alleviate this major concern, revealing that autophagic flux during KSHV latency depends on KapB and MK2.

We appreciated the importance of this reviewer's suggestion, and for that reason, prior to the current revision period, we tried several times to silence MK2 in primary human umbilical vein endothelial cells (HUVECs, hereafter, ECs) but were unsuccessful due to either lack of knockdown or incompatibility with cell growth (the silenced ECs stop proliferating and eventually die so we cannot complete the experiment). It is our many attempts at performing this experiment, and the fact that the original two first-authors have now left my lab, that have delayed resubmission of the current manuscript. After receiving an extension on the current revision, this summer we cloned, produced, and tested nine additional recombinant lentiviruses expressing shRNAs directed at MK2 and observed effective silencing of MK2 using two of these lentiviruses (sh1MK2 and sh2MK2). In the current revision of the manuscript, we show the effective silencing of MK2 when ECs were transduced with sh1MK2-expressing lentiviruses, as ECs expressing sh2MK2 died after being split and could not be used for subsequent infection.

To the current version of the manuscript, we now add a new 7-panel figure (Figure 8) using MK2-silenced ECs. In this figure we demonstrate effective silencing of MK2 (Fig 8A-B). We immunoblotted for phosphorylated hsp27 (Ser82) as a marker of MK2 activation (McCormick and Ganem, 2006, Corcoran et al. 2015) and show that latently infected cells have increased phosphorylation of hsp27 and therefore increased MK2 activity (Fig 8C-D). As expected, Ser-82 phosphorylation of hsp27 was significantly elevated in latent ECs that were infected with WT KSHV, but not in latent ECs infected by delB KSHV (Fig 8C); these data support the role of KapB in activating MK2 during latency. However, when MK2 was silenced, neither WT nor delB latently infected cells displayed significantly elevated phospho-hsp27 (Fig 8C-D). Importantly, in delB infected or uninfected cells, the reduction in phospho-hsp27 in the context of MK2 silencing was not significantly different from control cells whereas WT-infected cells showed a significant reduction in phospho-hsp27 in the context of MK2 silencing. These data confirm that KapB activates MK2 in the context of KSHV latency, solidifying the first step in the pathway *"KapB-->MK2-->Beclin 1 phosphorylation-->autophagy"* pathway noted above by the reviewer.

To connect MK2 activation to autophagic flux during KSHV infection, we silenced MK2 prior to infection and measured autophagic flux by immunoblotting for LC3-II accumulation after a time course BafA1 addition (Fig 8E-G). LC3-II accumulation was impaired in both uninfected and infected MK2-silenced cells, relative to control cells expressing a non-targeting shRNA (Fig 8F), indicating that MK2 contributes to autophagic flux in ECs during both uninfected and infected conditions. Because each of these experiments take a significant amount of time (cells are selected for two days after lentiviral transduction prior to the 4-day KSHV infection that ensures the establishment of latency, making the total time that the cells are silenced for MK2 >9 days), we were concerned that silenced cells adapted to MK2 loss, thereby altering the baseline autophagic flux of silenced cells compared to control cells. Indeed, when we silenced other important autophagy genes (e.g. Atg5) in this study, we preferred not to directly compare autophagic flux phenotypes between silenced and control cells because of the concern of autophagic pathway adaptation (see Figs 2, 6, 9, S2, and S4). Rather, data was compared within groups rather than between groups e.g. silenced uninfected to silenced infected cells. With this concern in mind, we compared LC3-II accumulation after BafA1 between different infection conditions all within MK2-silenced ECs and presented these analyses in Fig 8G.

We compared the following infection conditions: WT-infected vs uninfected, uninfected vs delB infected and WT infected vs delB infected and were able to derive some clear conclusions about the influential role of MK2 in autophagy during KSHV infection. First, LC3-II failed to accumulate in WT-infected MK2-silenced cells, and this was significantly different than the rate of accumulation in uninfected silenced cells. This suggests that during KSHV latency, MK2 is a significant contributor to the enhanced autophagic flux observed during infection; in its absence, autophagic flux is impaired. Second, there was no significant difference between LC3-II accumulation in delB infected cells compared to uninfected cells suggesting that during delB KSHV latency, MK2 activation does not contribute to autophagic flux. This is expected as we show delB KSHV infection fails to enhance the phosphorylation of hsp27 and therefore does not activate MK2 (Fig 8C). Third, in the context of MK2 silencing, LC3-II accumulation during WT latency was noticeably different than LC3-II accumulation during delB latency, further supporting the important role that KapB plays activating MK2 and autophagic flux during KSHV latent infection conditions. Taken together, these data support the second step in the pathway *“KapB-->MK2-->Beclin 1 phosphorylation-->autophagy”* pathway noted above by the reviewer.

In summary, with the addition of Figure 8, we have robustly answered the reviewer’s concerns using MK2 silencing and infection to show that: 1) MK2 is activated during WT but not delB KSHV latency and 2) using LC3-II accumulation as a measure of autophagic flux, MK2-silenced ECs displayed impaired autophagic flux during WT latency compared to uninfected or delB infected cells. In short, autophagic flux during KSHV latency depends on KapB and MK2.

Also, as suggested, a KapB deletion mutant that lacks MK2-interacting ability can somewhat alternatively address this issue since the authors successfully transfected KapB into HUVECs.

Regarding the reviewer’s suggestion to create a deletion mutant of KapB that lacks MK2-interacting ability – this is not feasible for the following reasons. The KapB protein sequence is comprised almost entirely of two sets of 23-amino acid direct repeat (DR) regions termed DR1

and DR2 and is predicted to be an intrinsically disordered protein. Previous work by Craig McCormick mapped the interaction between KapB and MK2 and defined the region of MK2 that binds KapB, but because KapB is a repetitive protein, no precise region within KapB was mapped for interaction with MK2 (loss of either set of DRs removes almost half of the total protein sequence). An *in vitro* pull-down assay showed that KapB binding to MK2 mapped to the DR2 repeat region of KapB, although the DR2-only KapB construct displayed much weaker interaction with MK2 than full-length KapB (McCormick and Ganem 2005). However, the ability of KapB to activate MK2 function, as measured by phosphorylation of its downstream effector hsp27 or stabilization of an ARE-containing reporter, required both DRs (McCormick and Ganem, 2006). Thus, prior effort did not identify a precise region of KapB responsible for activating MK2 that could be targeted by site-directed mutagenesis, a situation that is not unusual for a structurally plastic protein like KapB predicted to adopt multiple conformations.

2. Loss-of-function assay in already established KSHV latency. The authors argued that the use of KapB deficient KSHV virus for infection is a far superior approach. I agreed in previous version this truncated virus is a powerful tool. However, as Reviewer 3 also pointed, ".....why not use cells where KSHV latency is established like BCBL or iSLKs where they could directly compare the effect to the lytic cycle?". First, the authors did not show evidence that latency is established 96h postinfection. Second, I still think performing loss-of-function assays in already established KSHV latency are necessary to consolidate the conclusion. As shown above, the authors have no technique challenges to silence a gene in KSHV latency.

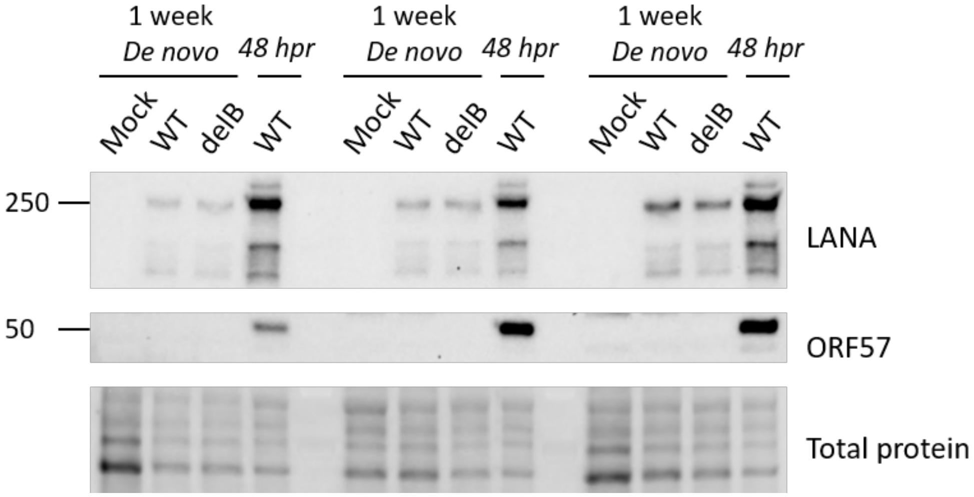

iSLK cells were infected with the indicated virus for one week to establish latency, then lysed for immunoblotting. As a positive control for expression of the lytic protein ORF57, parallel iSLK cells were reactivated for 48 hours using DOX. The independent experiments that are shown match the three independent experiments in Fig S7.

The experiments shown in Fig 7E of the prior version of the manuscript (Fig S7A of the current version) represent a latent infection of iSLK cells, as confirmed by immunoblotting infected cell lysates for LANA (latency marker) and ORF57 (lytic marker). Immunoblotting revealed that although LANA is expressed in latent iSLK cells infected with both WT and delB BAC16, ORF57 is only expressed after WT iSLK cells were induced to reactivate. These new data represent three independent experiments and have been added to the current version of the paper as Fig S7A.

Reviewer #2

1. According to the reviewer's comment, the authors performed a co-immunoprecipitation assay to examine whether NDP52 interacts with Dcp1a and the other PB protein Pat1b. However, the authors found that Dcp1a was not readily detected in the NDP52-immunoprecipitate (IP) complex. This is an unexpected result and different from the model proposed in the original manuscript, Instead, the authors found that Pat1b could be co-precipitated as shown in Fig. 8D. Unfortunately, the data was not appropriately presented; the author should show a full image of the GFP blot of Elution along with a molecular weight marker to verify that the control GFP alone was not co-precipitated with Flag-NDP52 (lane 1).

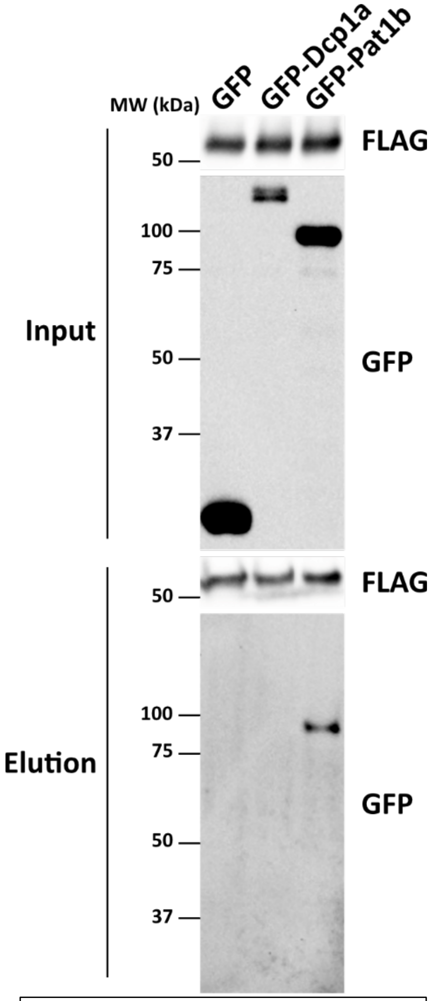

The co-immunoprecipitation experiment from prior Fig 8D (current Fig 10A with the full GFP elution blot shown.

If Dcp1a is expected to bind indirectly to NDP52 via Part1b as depicted in Fig. 8F, it should be readily detected in the NDP52-IP complex precipitated from the lysates of cells co-transfected with GFP-Pat1b.

Furthermore, the authors did not provide evidence yet to support "direct" interaction between NDP52 and Pat1b, for example, using a GST pull-down assay with purified proteins.

We have added the requested full image of the GFP elution blot and added molecular weight notations to alleviate the reviewer's concerns, as shown herein (Fig 8 in the prior version, Fig 10 in the current version). We agree with the reviewer that the experiment as shown does not provide support of a direct interaction between NDP52 and Pat1b and we apologize for the wording error. We have revised the wording describing our model shown in Fig 10C to ensure that we do not imply a direct interaction between NDP52 and Pat1b. We have also revised the graphic in Fig 10C, clarifying that based on its failure to immunoprecipitate with NDP52, Dcp1a protein is not likely to be a common constituent of the NDP52-Pat1b complex.

<u>Revised Fig 10C (model) Caption:</u>
"C: Model of PB disassembly induced by KSHV/KapB. KapB increases the phosphorylation of Beclin at Serine 90 to enhance autophagic flux during KSHV latency while Torin induces autophagic flux via the inhibition of mTORC1/2 and dephosphorylation of the ULK complex. Enhanced activation of autophagic flux by either mechanism results in PB disassembly that is dependent on the selective autophagy receptor, NDP52. In our model, we predict that NDP52 recruits a PB component(s) to the nascent autophagosome. Our top candidate for this recruitment is the PB scaffolding protein, Pat1b, as NDP52 co-immunoprecipitated with Pat1b and Pat1b protein level decreases after KSHV infection and Torin treatment. Steady state levels of Dcp1a

also decreased in response to KapB expression, KSHV infection or Torin treatment, suggesting that Dcp1a decreases may be mediated by its partial interaction with Pat1b despite observations that Dcp1a did not co-immunoprecipitate with NDP52. Autophagic degradation of Pat1b and Dcp1a, both key PB scaffolding proteins, lead to PB disassembly. "

2. The authors added new data to Fig. 6C, showing that torin-induced degradation of Pat1b was inhibited by NDP52 silencing. However, the degradation of its partner Dcp1a was not inhibited by NDP52 silencing. This is not in line with the model proposed by the authors that NDP52 and Pat1b could serve to deliver Dcp1a to the autophagosomes for degradation.

We have revised the wording used to describe our model in Lines 818-823 of the discussion, considering these reviewer's concerns. We have also revised the graphic and the wording in the Fig 10C caption as noted above, clarifying that Dcp1a is not significantly lost, is not likely to be a common constituent of the NDP52-Pat1b complex.

3. Recently, Calcoco1 has been known to bind to LC3 and serve as selective autophagy receptor (SAR) for the ER-phagy (Nthiga et. al., (2020) EMBO J. 39:e103649) and golgiphagy (Nthiga et. al., (2021) Autophagy 17:2051). In addition, TAX1BP1/Calcoco3 is known to act as SAR for xenophagy and aggrephagy. So, it was highly interesting to examine the activities of the NDP52 relatives in PB disassembly in addition to (or rather than) p62 and OPTN as performed in Fig. S4. This reviewer believes that it is important to examine if there is a functional redundancy for PB autophagy among the Calcoco family proteins.

The reviewer has made an insightful suggestion that NDP52 paralogs may also regulate KapB-mediated PB disassembly. NDP52 paralogs CALCOCO1 and CALCOCO3 have recently been established as important selective autophagy receptors, regulating the selective autophagy of ER, golgi, and protein aggregates (Nthiga et al. 2020, Nthiga et al. 2021, Shah et al. 2020). CALCOCO proteins have also been shown to be important regulators of virus infection, particularly of intrinsic immune responses controlled by RIG-I-receptors (Fan et al. 2020, Choi et al. 2021). We agree with the reviewer that a further investigation into these molecules in our system is warranted, but respectfully disagree with the reviewer that this investigation should be added to the current version of the manuscript. With ten multi-panel main figures and seven supplemental figures, our manuscript is already unwieldly, making the addition of a comprehensive analysis of the influence on two additional SARs on KSHV- and KapB-mediated PB disassembly beyond the scope of this study.

Moreover, prior to our study, compelling literature from non-infection systems existed to support a role for NDP52 as the major selective autophagy receptor to control PB turnover, whereas despite our searches, we have been unable to find any links to PBs or PB proteins in the literature for paralogs CALCOCO1 and CALCOCO3. Genetic manipulation of autophagic flux in yeast suggested that PB turnover was regulated by autophagy (Buchan et al.2013) while chemical inhibition of autophagy blocked PB accumulation (Hardy et al. 2017). Using overexpression of tagged PB proteins, one additional study demonstrated partial co-localization of GFP-tagged PB proteins and NDP52 (Guo et al. 2014). Our work expands significantly on these studies by using primary cells, incorporation of both genetic and chemical manipulation of autophagic flux and staining for endogenous PB proteins. In addition, to our knowledge, our work is the first example

of viral hijacking of autophagic turnover of a ribonucleoprotein granule, a manipulative strategy that leads to PB disassembly, thereby influencing the post-transcriptional regulation of cytokine mRNAs and the inflammatory environment associated with KSHV infection.

While we deem a comprehensive analysis of CALCOCO paralogs is beyond the scope of the current manuscript at this time, it will certainly shape a future direction of research in our lab.

4. The paper used the unusual quantification method to normalize and determine the relative intensities of immunoblot bands rather than using a loading control blot, such as Actin or GAPDH. It is still confusing. The authors did not mention a MW range of total protein blots used in normalization. In addition, it is very strange that the graphs of the relative band intensities were not changed even after the normalization by total protein blots. That is, the new graphs are exactly the same as the old ones. In addition, the authors did not provide the relative band intensities in some IB data. If normalized by total in Figs. 8B and S5A, the levels of Atg5 are unlikely reduced by shAtg5.

The authors did not describe how they performed the immunoblots for statistical analyses in Figs. 1A, 3E, 5B, 6C, 7B, 7D, 7E, 8C, and 8E. Where were they from different blots of the same lysates or each blot from three to four independent experiments?

We apologize to the reviewer for the confusion. Our lab performs densiometric analysis of protein bands following immunoblotting using a method that takes advantage of stain-free gel and blot technology offered by BioRad (https://www.bio-rad.com/fr-ca/applications-technologies/stain-free-imaging-technology?ID=NZ0G1815). This method is superior to standard methods of immunoblotting for abundant housekeeping proteins like actin/GAPDH and is rapidly becoming widely adopted in the field. This method permits visualization of total protein without the need for Coomassie Blue or Ponceau S staining and still uses total protein as basis for normalization. A trihalo compound is present in TGX Stain-Free premixed acrylamide solutions that covalently binds to tryptophan residues within the electrophoresed protein when activated with UV light. Activation of the trihalo compounds in the gel adds 58 Da moieties to available tryptophan residues and is required for protein visualization. This allows protein detection (with a stain-free enabled imager) in the gel both before and after transfer, as well as total protein detection on a blot using wet PVDF membranes. The sensitivity of the stain-free system is comparable to staining with Coomassie Blue for proteins with a tryptophan content >1.5% and superior to Coomassie for proteins with a tryptophan content >3%. This stain-free technology is also compatible with immunoblotting applications. After images of total protein and our protein of interest have been obtained, our quantification method ensures that band intensities are in the linear range for both the protein of interest and the total protein visualized using stain-free technology using published methods (Taylor et al 2013, Taylor and Posch 2014). Briefly, the band of interest is normalized to the total protein signal from its matched lane. This approach eliminates inaccurate results that occur when a weak signal for the protein of interest is normalized to an intense or saturated signal from a highly expressed housekeeping gene such as actin/GAPDH.

In addition, we would like to clarify for the reviewer that the graphs of band intensities did not change in the revised version because the method of normalization did not change. In all versions

of this manuscript, densiometric measure of individual protein bands were normalized to total protein as described above. The only thing that was altered between earlier versions of the figures and the most recent version was the y-axis label which we altered as an attempt to make our quantitation method clearer. Finally, in all statistical analysis of densitometry results, we quantified bands from three (or more) independent experiments; these are not technical replicates or triplicate immunoblots from the same experiment. These additional technical details have been added the methods of the newly revised version of the manuscript.

5. The comments raised by the reviewer in Fig. 3A (new Fig. 4A) were simply 1) to provide the statistical evaluation (either ns or p-value) of the expression of IL-6 between control and KapB cells at 0 h, 2) to statistically identify the effects of the conditional media in either control or KapB cells on cytokine expression, and 3) to examine the combined effects of the conditional media and torin in cytokine expression in Fig. 3B.

The authors mentioned that KapB expression and transcriptional stimulus (the conditional media) significantly enhanced IL-6 and IL-1 beta levels. However, it is unlikely that IL-1 beta expression in KapB cells is enhanced by addition of the media for 6 h, that is, no effect of the conditional media.

We have added the missing statistical evaluations to the graphs in Fig 4A as requested by the reviewer. We apologize for the oversight. In addition, we have modified the interpretation of these data as suggested by the reviewer in Lines 429-468.

Additional interpretation of Fig 4A-B added:
"We then examined levels of some endogenous ARE-containing RNA transcripts in KapB-expressing cells (Fig 4A). After KapB expression, IL-1β mRNA was significantly elevated compared to equivalently treated controls both before and after conditioned media treatment; however, the IL-6 was transcript was significantly increased only after conditioned media (Fig 4A). We interpret these data as follows. First, transcriptional stimulus did not further enhance IL-1β mRNA levels, as the steady-state of this mRNA was already elevated in the context of KapB expression. Second, KapB-mediated elevation of IL-6 transcript could be detected only after transcriptional stimulus, suggesting that in the absence of transcriptional upregulation IL-6 mRNA levels were not sufficient to benefit from the absence of PB turnover. Together, these data suggest that some cytokine transcripts respond to KapB-mediated PB disassembly with elevated levels either at baseline or upon transcriptional induction that we suggest is a result of reduced transcript decay in PBs (Fig 4A). In addition, treatment with Torin promoted the enhanced steady-state levels of IL-1β and COX-2 (Fig 4B), suggesting that inducing autophagy and PB disassembly by an alternative mechanism also reduced the decay of some ARE-mRNA transcripts. This suggests that cytokine mRNAs that shuttle to PBs are likely to respond to diverse stimuli that upregulate autophagic flux."

Minor Issues

Reviewer #1:

3. Fig 4A (new Fig 5A). Again, the current data show that silencing ATG5 upregulates Dcp1a, but it is not correct that silencing ATG5 restores KapB-mediated Dcp1a loss.

We thank the reviewer for pointing out this discrepancy in our language. Our data showed silencing Atg5 restored Dcp1a protein levels in both KapB-expressing cells and control cells (Fig 5D). This suggests that Dcp1a protein turnover is subject to autophagic regulation in all cells. Since KapB expression further decreased Dcp1a protein levels (Fig 5D), in our model we propose that KapB accelerates this autophagic turnover. We have corrected this statement in the manuscript, Lines 508-511.

Additional interpretation of Fig 5D added:
"Silencing the autophagy gene, *Atg5*, resulted in a reversal of Dcp1a loss in both KapB and control cells (Fig 5D), suggesting that autophagy is required for the turnover of Dcp1a and that KapB accelerates this autophagic turnover. "

4. "If basal autophagy is low, there should be equal levels of LC3-I and LC3-II in the cell". This statement is incorrect. LC3-II is an autophagic cleavage product of LC3-I. If the basal level of autophagy is low, LC3-II level should be low (could be undetectable by IB). So, again, in Fig 1A, if KapB induces autophagy, we should see elevated LC3-II level at 0 h in KapB vs Control samples (Labe 1 vs Lane 3), and LC3-II should be low at 0h in Control sample (lane 1) if the basal level of autophagy in HUVECs is low.

We thank the reviewer for the correction. We do agree that our original statement was confusing and have removed any such similar statement from the manuscript. To clarify our meaning, after LC3-I is cleaved and lipidated, it associates with autophagosomes. As these autophagosomes fuse with lysosomes, LC3-II is degraded along with the cargo contained within the autophagosome. From a practical perspective, this means that when autophagic flux is increased at all steps throughout the pathway, such as when autophagy induction occurs at early initiation stages (as we propose for KapB), although more LC3-II is *produced*, more LC3-II is also *degraded* by the final degradative step in the pathway. This means that in the absence of a drug that blocks autophagic degradation (such as BafA1 used herein), it is difficult to determine if a protein elevates autophagic flux without the pairwise comparison of LC3-II accumulation with and without BafA1 treatment. We respectfully challenge the reviewer's comment that LC3-II levels should be demonstrably higher after KapB expression versus control cells *in the absence of BafA1 treatment* as this is more often representative of a blockage in the degradative step of autophagic flux that permits the accumulation of LC3-II, which is not what we have proposed. A comparison of LC3-II between +/- BafA1 conditions is required to differentiate between these two possibilities. Throughout the manuscript, we consistently analyzed autophagic flux in this manner (Figs 1, 7, 8, S7), always including BafA1 treatment when we measure LC3 puncta size or performing a time course of BafA1 treatment when we measured LC3-II accumulation by immunoblotting.

The analysis we have performed, and the explanations above, are consistent with the most recent guidelines on measuring autophagic flux by Klionsky et al. 2021. Taken from these guidelines are the following statements: "The relevant parameter in LC3 assays is the difference in the amount of LC3-II in both the presence and absence of saturating levels of inhibitors, which can

be used to examine the transit of LC3-II through the autophagic pathway; if flux is occurring, the amount of LC3-II will be higher in the presence of the inhibitor." "Increased levels of LC3-II in the presence of lysosomal inhibition or interfering with autophagosome-lysosome fusion alone (e.g., with bafilomycin A_1), may be indicative of greater induction and cargo sequestration, but to assess whether a particular treatment alters complete autophagic flux through substrate digestion, the treatment plus bafilomycin A_1 must be compared with results obtained with treatment alone as well as with bafilomycin A_1 alone. … If the treatment by itself increases LC3-II levels, but the treatment plus bafilomycin A_1 does not increase LC3-II levels compared to bafilomycin A_1 alone, this may indicate that the treatment induced a partial block in autophagic flux. Thus, a treatment condition increasing LC3-II on its own that has no difference in LC3-II in the presence of bafilomycin A_1 compared to treatment alone may suggest a complete block in autophagy at the terminal stages."

5. "We tried to immunoblot for vFLIP to detect its expression using two different commercial antibodies, without success". This is an unacceptable excuse. KSHV vFLIP has been well studied and IB results are presented in tones of papers.

In response to the prior reviewer comment, *"Primary KSHV infection induces autophagy despite vFLIP: have the author check vFLIP expression in these cells?",* we expressed that after several purchases, we had failed to identify a successful commercial antibody that could detect vFLIP protein on an immunoblot. We expand on this response here.

Our recent manuscript (Kleer et al 2022) described the creation of a panel of kaposin-deficient KSHV BAC16 recombinant viruses. For each of our recombinant viruses, including WT BAC16 and delB BAC16 used in the current manuscript, we performed next-generation sequencing of the entire bacmid and showed that there were no changes to any region in the KSHV genome, save for those intended changes in the kaposin locus. These data are presented in Fig 2 of Kleer et al 2022 (https://doi.org/10.1128/jvi.01560-21). Moreover, we also showed that delB BAC16 retained the ability to cause EC spindling in the absence of KapB expression, a functional readout for the expression of vFLIP in the context of our KSHV infection model (Grossman et al. 2006). These data are presented in Fig 10 of Kleer et al 2022. (https://doi.org/10.1128/jvi.01560-21).

vFLIP is translated from a polycistronic transcript that also encodes v-Cyclin (Othman et al. 2014). vFLIP expression is low in infected cells due to inefficient codon usage, but this is deemed advantageous to the virus, as codon optimization decreases the efficiency of lytic reactivation (Bellare et al. 2015). The low level of translation of vFLIP during latency combined with the rapid turnover of the protein (Chang et al., 2006, Chmura et al. 2017, Choi et al. 2018) makes virally encoded vFLIP very difficult to detect using many available commercial antisera. In response to the reviewer's statement that vFLIP is detected in tons of paper, we performed a PubMed search and examined the recent KSHV literature for some examples. Of the KSHV papers that showed vFLIP expression by immunoblotting, the majority did so by immunoblotting for a tagged version of vFLIP (see list).

Papers that detect vFLIP using antisera to tagged vFLIP:
https://journals.asm.org/doi/10.1128/jvi.00226-11

Alkharsah et al, JVI, 2011.
https://www.ncbi.nlm.nih.gov/pmc/articles/PMC2802862/
Lee et al, Nat Cell Biol, 2009.
https://www.sciencedirect.com/science/article/pii/S1931312812000261?via%3Dihub
Leidel et al, Cell Host and Microbe, 2011.
https://www.ncbi.nlm.nih.gov/pmc/articles/PMC1200255/
Chugh et al, PNAS, 2005.
https://link.springer.com/article/10.1007/s13402-022-00701-9
Mohanty et al, Cellular Oncology, 2022.
https://www.nature.com/articles/s41388-019-0726-5
Nakayama et al, Oncogene, 2019.
https://www.ncbi.nlm.nih.gov/pmc/articles/PMC6748093/
Ding et al, Cell Death Differ, 2019.
https://www.sciencedirect.com/science/article/pii/S0042682218302095?via%3Dihub
Yang et al, Virology, 2018.
https://www.ncbi.nlm.nih.gov/pmc/articles/PMC5271570/
Chmura et al, Virology, 2018.
https://www.ncbi.nlm.nih.gov/pmc/articles/PMC4636184/
Choi et al, Plos Pathogens, 2015.
https://www.ncbi.nlm.nih.gov/pmc/articles/PMC4473549/
Bellare et al, JVI, 2015.
https://www.ncbi.nlm.nih.gov/pmc/articles/PMC4301867/
Ballon et al, PLOS Pathogens, 2015.

Papers that describe vFLIP function but do not immunoblot:
https://doi.org/10.1371/journal.ppat.1009600
Chen et al 2021. PLoS Pathog 17(12): e1009600.

Papers that appeared to detect untagged vFLIP via immunoblot but information on the antibody was lacking:
https://www.ncbi.nlm.nih.gov/pmc/articles/PMC9163697/
Bagneris et al. 2021. J. Biol. Chem.
*This manuscript appears to immunoblot for vFLIP after co-immunoprecipitation; however, no information about the antibody is provided.

https://www.ncbi.nlm.nih.gov/pmc/articles/PMC5963799/
Choi YB, Choi Y, Harhaj EW (2018) Peroxisomes support human herpesvirus 8 latency by stabilizing the viral oncogenic protein vFLIP via the MAVS-TRAF complex. PLoS Pathog 14(5): e1007058. https://doi.org/10.1371/journal.ppat.1007058
* In this manuscript, it appears that some immunoblotting is performed against authentic vFLIP (although most is tagged-vFLIP). However, in the supplementary information listing the source of the antibodies, the reference listed for the polyclonal rabbit anti-vFLIP is Chumara et al 2017 which, as noted above, only used antisera to tagged vFLIP.

Papers that detect untagged vFLIP via immunofluorescence:
Sander et al. 2008. J. Virol. 82(4) 1908–192
doi:10.1128/JVI.01716-07

*To confirm the subcellular localization of tagged vFLIP, the authors of this study describe the production of antisera directed to the authentic vFLIP protein. “The K13 (clone 4C1) and the K10.5 (clone 3G7) monoclonal antibodies were produced by immunization of LOU/C rats with His-tagged purified recombinant K13 (full-length) protein or K10.5 (N-terminal 98-amino-acid fragment) protein (50 g each) according to a previously described procedure.” We reached out to this lab in summer 2021 but have not yet received vFLIP antibody to try in our experiments.

Email correspondence requesting vFLIP antisera:
Dear Dr. Stürzl,

I am writing to you from the University of Calgary in Canada, where my lab studies KSHV. We are particularly interested in the kaposin region of the virus, but because some of the phenotypes elicited by KapB overlap with those induced by vFLIP, we have initiated a project where we co-express these two viral genes in primary endothelium. As you know, vFLIP expression is very difficult to detect. We have tried tagging vFLIP with two different fluorescent proteins (cells typically die after the lentiviral transduction) , and commercial antibodies have also brought us limited success. Although we select transduced cells to make sure they have the lentivirus, and the cells do spindle so we know vFLIP is expressed, this readout does not work for our co-expression (double transduction) scenario. At the recent KSHV meeting my student, Lena (copied) saw a presentation from a group that used your monoclonal Ab for vFLIP (described in Sander et al JVI 2008). We are writing to ask if you would consider sharing a small amount of this Ab in order that we can verify expression of authentic vFLIP.
I am happy to share more information about our research interests. Thanks for considering my request.

Best regards,
Jenn

Stürzl, Michael <Michael.Stuerzl@uk-erlangen.de>
[⚠EXTERNAL]

Dear Jennifer,

could you please inform me which group presented the poster and which clone they used (clone 4C1?).
Best,
Michael

In summary, although we appreciate this comment from the reviewer, detection of untagged vFLIP clearly remains a significant issue in the KSHV field. If the reviewer could suggest a commercially available vFLIP antibody that works in their hands to detect virally encoded vFLIP during KSHV latency, we would be very receptive to this information.

6. Since the authors failed to show KSHV induces NDP52, which is expected in that KSHV activates NRF2 that induces NDP52, the possibilities should be discussed.

We agree. It was initially surprising to us that KSHV infection did not enhance increase steady-state levels of NDP52 protein, as others have shown that KSHV infection increases the activity of the Nrf2 transcription factor and NDP52 is an Nrf2-responsive gene (Gjyshi et al. 2015). We have added additional interpretation and discussion of our data to reflect this observation. However, it not unusual for a selective autophagy receptor to be regulated post-transcriptionally, as the ability of these molecular bridges to induce autophagosome engulfment of target cargo

must be a rapid response to changes in the cell environment, making transcriptional upregulation less likely. In addition, increased utilization of NDP52 as a SAR would, by definition, enhance its own protein turnover concomitant with autophagy induction. This feedback loop phenomenon has been nicely characterized for the SAR, p62/SQSTM1, which has been shown to bind to the Nrf2 negative regulator, KEAP1 using a KEAP-interacting region adjacent to its LIR domain. Binding of p62 oligomers simultaneously to KEAP1 and LC3 induces the autophagic degradation of KEAP (and p62 concomitantly), activating Nrf2 to maintain homeostatic p62 levels (Jain et al. 2010).

There are several examples of post-translational modification, most commonly phosphorylation, of SARs including NDP52 that alter SAR function (Fan et al. 2020, Choi et al 2021, Fu et al., Heo et al., Thurston et al., Wild et al., Richter et al., Matsumoto et al.). Studies that examine if post-translation modification(s) of NDP52 are elicited by KapB or KSHV infection to alter NDP52-dependent PB disassembly are a planned future direction of research in our lab. We will also investigate potential modification of PB proteins that occur in response to KapB/KSHV that may likewise enhance their association with NDP52.

Revisions to discussion section:
"We show herein that KapB also upregulates autophagic flux, and that KapB is necessary for the enhanced autophagic flux we observe after KSHV infection of primary endothelial cells. Though these data are consistent with autophagy enhancement observed in latent B-lymphocytes (BCBL-1 cells) (127), they are discordant with a report that KSHV infection dampens autophagic flux and mitophagy (128). It is not unusual for viruses to display such a push/pull relationship; during coxsackievirus B3 infection, p62 is cleaved by a viral protease to limit its antiviral action while an NDP52 cleavage product retains proviral activity (36). Such studies lend support to the complex manipulation of selective autophagy pathways that emerging for KSHV infection. We considered the possibility that KSHV products may cooperate to promote autophagy during latency via dysregulation of the cellular transcription factor, Nrf2. Nrf2 activity is upregulated by the viral protein vFLIP after *de novo* infection of ECs and during KSHV latency (129,130) and Nrf2 is responsible for the transcriptional activation of several antioxidant response genes as well as NDP52 (131–133). However, we did not observe KSHV-mediated increases in NDP52 protein after infection, making it more likely that KapB-mediated enhancement of NDP52-dependent selective autophagy of PBs proceeds via post-translational regulation of NDP52 and/or PB components (129,130)."

New critiques:
7. Canonical vs noncanonical autophagy. The authors make inconsistent claims on canonical vs noncanonical autophagy forms induced by KapB. For example, "KapB failed to activate autophagic flux by canonical pathways that are used by Torin". "Canonical autophagy is required for KapB-mediated increases in ARE-containing cytokine transcripts". Please clarify what is the exact form of autophagy and what are their differences.

We thank the reviewer for pointing out the inconsistent language used in our manuscript. In the last few years, the autophagy field has moved towards eliminating use of the term 'canonical' as our recognition of the complex recognition of autophagy increases. We have removed the word 'canonical' from our manuscript and replaced it with more specific language in each case.

8. Line 159: Subtitle: KapB-mediated PB disassembly requires Atg5 and Atg14. Line 322: Subtitle: Atg5 and NDP52 are required for KSHV-mediated PB disassembly. Reorganization is needed to put ATG5 data together.

We appreciate this reviewer's comment and their dedication to a consistent pattern of manuscript organization. We seriously considered the reviewer's suggested re-organization of our data, and we mapped out how this could be achieved together in several sessions at the white board. Upon completion of this exercise, we concluded that the narrative was not improved by making the suggested changes. Our manuscript combines complementary reductionist (KapB expression), chemical (Torin) and infection (KSHV vs delB) approaches to study autophagic flux as it pertains to PB disassembly during KSHV latency. Clearly, this provides many options for different kinds of organization. In the end, we have chosen to keep the manuscript organized as is for clarity, leading with KapB ectopic expression approaches and following with infection systems.

References

Boyle et al. 2019. CALCOCO2/NDP52 initiates selective autophagy through recruitment of ULK and TBK1 kinase complexes. Autophagy. 15(9):1655-1656.

Buchan et al. 2013. Eukaryotic stress granules are cleared by autophagy and Cdc48/VCP function. Cell 153, 1461–1474

Chiang et al. 2013. Phosphorylation of mRNA Decapping Protein Dcp1a by the ERK Signaling Pathway during Early Differentiation of 3T3-L1 Preadipocytes. PLoS ONE, 8(4).

Choi et al. 2021 Biorxiv. Phosphorylation of the selective autophagy receptor TAX1BP1 by canonical and noncanonical IκB kinases promotes its lysosomal localization and clearance of MAVS aggregates. doi: https://doi.org/10.1101/2021.01.07.425702

Corcoran et al. 2015. Viral activation of MK2-hsp27-p115RhoGEF-RhoA signaling axis causes cytoskeletal rearrangements, p-body disruption and ARE-mRNA stabilization. PLOS Pathog. 11: e1004597.

Fan et al. 2020 The role of autophagy and autophagy receptor NDP52 in microbial infections. Int J. Mol. Sci. *21*(6)

Fu et al 2018 Mechanistic insights into the interactions of NAP1 with the SKICH domains of NDP52 and TAX1BP1. Proc Natl Acad Sci 115(50):E11651–E60.

Grossman et al. 2006. Activation of NF-kB by the latent vFLIP gene of Kaposi's sarcoma-associated herpesvirus is required for the spindle shape of virus infected endothelial cells and contributes to their proinflammatory phenotype. J. Virol. 80(14):7179-85.

Guo et al. 2014 Autophagy supports genomic stability by degrading retrotransposon RNA. Nat. Commun. 5, 5276

Hardy et al. 2017. Regulation of epithelial-mesenchymal transition and metastasis by TGF-β, p bodies, and autophagy. Oncotarget 8, 103302–103314

Heo et al. 2015. The PINK1-PARKIN Mitochondrial Ubiquitylation Pathway Drives a Program of OPTN/NDP52 Recruitment and TBK1 Activation to Promote Mitophagy. Mol Cell. 60(1):7–20.

Jain et al. 2010 p62/SQSTM1 is a target gene for transcription factor NRF2 and creates a positive feedback loop by inducing antioxidant response-element-driven gene transcription, J. Biol. Chem. 285(29): 22576-91

Kleer et al 2022. A panel of KSHV mutants in the polycistronic kaposin locus for precise analysis of individual protein products. J. Viol. 96(5):e0156021.

Klionsky et al. 2021. Guidelines for the use and interpretation of assays for monitoring autophagy. Autophagy 17(1):1-382.

Matsumoto et al 2011 Serine 403 phosphorylation of p62/SQSTM1 regulates selective autophagic clearance of ubiquitinated proteins. Mol Cell. 44(2):279–89.

McCormick and Ganem 2005 The Kaposin B Protein of KSHV Activates the p38/MK2 Pathway and Stabilizes Cytokine mRNAs. Science. 307: 739–741.

McCormick and Ganem 2006. Phosphorylation and Function of the Kaposin B Direct Repeats of Kaposi's Sarcoma-Associated Herpesvirus. J. Viol. 80(12): 6165–6170.

Othman et al. 2014 Functional analysis of Kaposi's sarcoma-associated herpesvirus vFLIP expression reveals a new mode of IRES-mediated translation. RNA. 20(11):1803-14.

Pilli et al. 2012 TBK-1 promotes autophagy-mediated antimicrobial defense by controlling autophagosome maturation. Immunity. 37(2):223–34.

Richter et al. 2016 Phosphorylation of OPTN by TBK1 enhances its binding to Ub chains and promotes selective autophagy of damaged mitochondria. Proc Natl Acad Sci 113(15):4039–44.

Shah et al. 2020. Loss of TAX1BP1-directed autophagy results in protein aggregate accumulation in the brain. Mol Cell. 80(5): 779-95 e10.

Taylor et al. 2013. A defined methodology for reliable quantification of Western blot data. Mol. Biotechnol. 55:217–226.

Taylor and Posch 2014.The design of a quantitative western blot experiment. Biomed Res Int 2014:361590-8.

Tenekeci et al. 2016. K63-Ubiquitylation and TRAF6 Pathways Regulate Mammalian P-Body Formation and mRNA Decapping. *Molecular Cell*, *62*, 943–957.

Thurston et al. 2009 The TBK1 adaptor and autophagy receptor NDP52 restricts the proliferation of ubiquitin-coated bacteria. Nat Immunol. 10(11):1215–21.

Wani et al. 2015. Regulation of autophagy by post-translational modification. Lab Invest. 95(1): 14–25.

Wild et al. 2011 Phosphorylation of the autophagy receptor optineurin restricts Salmonella growth. Science. 333(6039):228–33.
