# Supplementary material for: Kaposi’s sarcoma-associated herpesvirus (KSHV) utilizes the NDP52/CALCOCO2 selective autophagy receptor to disassemble processing bodies
Source: PLoS Pathog. 2023 Jan 12;19(1):e1011080. doi: 10.1371/journal.ppat.1011080 (PMC9876383; doi:10.1371/journal.ppat.1011080)
Supplement: Attachment — Submitted filename: Response to Reviewers Final.pdf [file ppat.1011080.s011.pdf]

Response to Reviewers

Minor Concerns:

Reviewer #1:

Still think these two sections should be re-organized regarding ATG5. It is confusing in the current format
1. KapB-mediated PB disassembly requires Atg5 and Atg14
2. Atg5 and NDP52 are required for KSHV-mediated PB disassembly

We appreciate the reviewer's comment about the organization of our manuscript. We took their recommendations very seriously and re-organized the data as suggested, both dynamically at the white board in a group meeting, as well as in the written manuscript. Because there is a tremendous amount of work summarized in this paper, one of our primary goals is to present the data with great clarity and transparency. Unfortunately, in the end, all co-authors agreed that the proposed re-organization did not improve clarity. This is because the initial studies used a reductionist approach, employing KapB expression or a chemical (Torin) to determine the influence of autophagic flux and selective autophagy on PB disassembly. Using these 'simpler' approaches, we first identified the autophagy genes that could be silenced in primary endothelial cells (without killing them) and then determined which ones were required for KapB- and Torin-mediated PB loss. These preliminary analyses were quite extensive, but they were worth it, because it allowed us to narrow our focus to study only the most relevant autophagy/selective autophagy receptor genes in the context of the more cumbersome KSHV infection experiments using WT and delB viruses.

In summary, our extensive analysis of the requirement for autophagy in PB disassembly in the context of KapB expression, Torin treatment, and KSHV latency provides several options for different kinds of organization. In the end, we chose to keep the current manuscript structure for optimal clarity, leading with KapB ectopic expression approaches that characterize the PB disassembly requirement for selective autophagy in detail, and following with infection systems to confirm that in the context of KSHV, the same pathways are engaged. We think that a change to the order of data presentation would lead to discontinuities and lack of symmetry that would make this paper more difficult to read. We thank the reviewer for their understanding our of choice.

Reviewer #2:

The #3 concern was whether the other NDP52 paralogs, Calcoco1 and Calcoco3, are involved in PB disassembly. This reviewer thought that the experiment would be straightforward when the authors have the cell lines that are knocked down or out for the genes. The new data could be added to Fig. S4 along with the data of p62 and OPTN. Nonetheless, I understand and agree to the authors' response.

The experiments presented in this manuscript are extremely technically challenging. We use primary endothelial cells throughout the paper because they are the most biologically relevant

cell type. The endothelial cells used for these experiments can be maintained only for a limited passage number as changes in cell morphology and phenotype are detected with extended culturing (Liao et al. 2014). We never use HUVECs past passage 8, which is quite conservative. However, these cells are hard-to-transfect and require that overexpression is achieved by separate lentiviral transduction for each experiment. Therefore, many of our experiments required sequential transduction of primary cells with i) a shRNA-expressing population of lentiviruses and ii) a KapB-expressing or control population of lentiviruses. For each independent experiment, a different passage of HUVECs and a separate transduction is used, an approach that can increase variability between data points but overall provided the most accurate and robust analysis of our phenotype. We have not used any stable cell lines in this paper except for HeLa Tet-Off cells used for the luciferase reporter assay experiments.

We are grateful to the reviewer for their acknowledgement that RNA silencing of NDP52 paralogs CALCOCO1 and CALCOCO3 in HUVECs may not be trivial. For that reason, we have not performed additional analyses of the influence of NDP52 paralogs on PB disassembly, but we do agree that these are important questions to pursue in future studies.

The concerns in #5 were 1) to provide the statistical evaluation (either ns or p-value) of the expression of IL-6 between control and KapB cells at 0 h….

We thank the reviewer for clarifying their request and apologize for our misunderstanding. In our prior response, we completed the first request and address the other requests here.

2) to statistically identify the effects of the conditional media in either control or KapB cells on cytokine expression, and….

We have performed the additional statistical analysis as per the reviewer's second request, comparing cytokine transcript levels before and after conditioned media treatment within groups (control or KapB expression). The modified graphs are now in Fig 4A. We thank the reviewer for this good suggestion.

3) to examine the combined effects of the conditional media and torin in cytokine expression in Fig. 4B. The authors have not responded to the second and third concerns. The last concern was about the relationship between transcription and autophagy regulation of the expression of the cytokine genes.

Torin inhibits mechanistic target of rapamycin complexes 1 and 2 (mTORC1 and mTORC2), causing dephosphorylation of Atg13 and Ulk1/2 and robust induction of autophagy (Alers 2012). We have demonstrated that KapB stimulates autophagic flux using a mechanism that is distinct from autophagy regulation by Torin; specifically, KapB acts like constitutively active MK2 to phosphorylate the autophagy regulator Beclin at Serine 90. When incorporating Torin into our analyses of PB disassembly, we asked the following question: does a different mechanism of autophagy induction cause PB disassembly and increase ARE-containing mRNA transcripts? In Fig 3A-B, we showed that Torin induced PB disassembly. In Fig 4B, we demonstrated that Torin-treated HUVECs displayed elevated levels of mRNA encoding cytokines IL-1β, COX-2 and IL-6 but not elevation of the RNA transcript for the chemokine, CXCL8. These results

answered our research question, supporting a requirement for the induction of autophagy in PB turnover leading to increases in steady-state levels of certain cytokine mRNAs. We also note that these data are consistent with the findings of others in the field, including a recent investigation that tested the combined effects of Torin treatment plus the cytokine TNF (used to induce cytokine gene transcription). These investigators showed TNF treatment further increased inflammatory cytokine transcripts that were also induced by Torin alone (Chen et al. 2021, Peng et al. 2020). We chose not to duplicate these experiments and combine the analysis of Torin-induced cytokine changes with conditioned media treatment.

And the following new text is not easy for me to follow. It'd be nice to rewrite.
"We then examined levels of some endogenous ARE-containing RNA transcripts in KapB expressing cells (Fig 4A). After KapB expression, IL-1b mRNA was significantly elevated compared to equivalently treated controls both before and after conditioned media treatment; however, the IL-6 was transcript was significantly increased only after conditioned media (Fig 4A). We interpret these data as follows. First, transcriptional stimulus did not further enhance IL-
1b mRNA levels, as the steady-state of this mRNA was already elevated in the context of KapB expression. Second, KapB-mediated elevation of IL-6 transcript could be detected only after transcriptional stimulus, suggesting that in the absence of transcriptional upregulation IL-6 mRNA levels were not sufficient to benefit from the absence of PB turnover. Together, these data suggest that some cytokine transcripts respond to KapB-mediated PB disassembly with elevated levels either at baseline or upon transcriptional induction that we suggest is a result of reduced transcript decay in PBs (Fig 4A). In addition, treatment with Torin promoted the enhanced steady-state levels of IL-1β and COX-2 (Fig 4B), suggesting that inducing autophagy and PB disassembly by an alternative mechanism also reduced the decay of some ARE-mRNA transcripts. This suggests that cytokine mRNAs that shuttle to PBs are likely to respond to diverse stimuli that upregulate autophagic flux."

We apologize for the unclear nature of this section of the manuscript. We have significantly reworded this section of the results (p.13-14). The reworded text is also found below.

Amended Text:

"PBs are nodes of post-transcriptional mRNA regulation and microscopically visible PBs are associated with ARE-mRNA decay or suppression (59,60,62,63,96–98). However, under basal conditions, there is limited transcription of most ARE-containing cytokine or chemokine transcripts, making their measurement difficult. Rather than using an irrelevant inflammatory stimulus such as lipopolysaccharide to activate transcription of ARE-containing cytokines, we chose to activate their transcription in a biologically relevant manner by mimicking the inflammatory environment of the KS lesion. KapB-expressing or control HUVECs were treated with 0.22 µm-filtered conditioned media from KSHV latently infected cells, expected to contain inflammatory cytokines, e.g. TNF that would then further activate cytokine gene transcription (99). We determined the RNA level of endogenous ARE-containing transcripts in KapB-expressing cells before and after the conditioned media treatment and compared these to equivalently treated control HUVECs (Fig 4A). In the context of KapB expression, IL-1b mRNA was significantly elevated compared to equivalently treated controls before and after conditioned

media treatment (Fig 4A). These data suggest that this ARE-containing transcript is subject to constitutive decay in PBs that is alleviated by KapB. Moreover, it suggests that KapB-mediated PB disassembly enhances the RNA levels at baseline as well in the context of inflammatory stimuli typical of the KS microenvironment. IL-6 transcript levels significantly increased in KapB-expressing cells after but not before conditioned media treatment (Fig 4A). These data suggest that the IL-6 transcript requires an inflammatory transcriptional stimulus (e.g. KS microenvironment) combined with KapB-mediated PB disassembly for a significant increase. RNA transcripts encoding COX-2 and the chemokine CXCL8 were not significantly elevated by conditioned media treatment alone, nor were they elevated by KapB expression alone, or by the combination of media treatment and KapB (Fig 4A). These data suggest that additional factors contribute to elevated RNA levels of these ARE-containing RNA transcripts. The observation that different cytokine RNA transcripts respond differently to alterations to PB dynamics was also made by others (115). Together, these data suggest that some cytokine transcripts respond to KapB-mediated PB disassembly with elevated levels either at baseline or upon transcriptional induction, an effect that likely results from reduced transcript decay in PBs (Fig 4A). In addition to KapB, we also treated HUVECs with Torin. This treatment enhanced steady-state levels of IL-1β and COX-2 (Fig 4B), suggesting that the induction of autophagy by an alternative mechanism that also culminated in PB disassembly was sufficient to elevate certain ARE-mRNA transcripts. This suggests that cytokine mRNAs that shuttle to PBs are relieved from constitutive turnover by stimuli that upregulate autophagic flux and cause PB disassembly. "

References

Alers et al. 2012. Role of AMPK-mTOR-Ulk1/2 in the Regulation of Autophagy: Cross Talk, Shortcuts, and Feedbacks. Mol. Biol. Cell. 1:2-11

Chen et al. 2021 mTOR inhibition promotes pneumonitis through inducing endothelial contraction and hyperpermeability. American Journal of Respiratory Cell and Molecular Biology. 65(6) 646-657.

Liao et al. 2014. Effects of long-term serial cell passaging on cell spreading, migration, and cell-surface ultrastructures of cultured vascular endothelial cells. Cytotechnology. 66(2):229-238.

Peng et al. 2020. IRF-1 mediates the suppressive effects of mTOR inhibition on arterial endothelium. J Mol Cell Cardiol. 140: 30–41.
